# Body Fat-Reducing Effects of Whey Protein Diet in Male Mice

**DOI:** 10.3390/nu15102263

**Published:** 2023-05-10

**Authors:** Kimitaka Nakazaki, Nobuhiko Nagano, Daichi Katayama, Shoichi Shimizu, Kengo Matsuda, Wataru Tokunaga, Ryoji Aoki, Kazumasa Fuwa, Ichiro Morioka

**Affiliations:** Department of Pediatrics and Child Health, Nihon University School of Medicine, Tokyo 173-8610, Japan; nakazaki.kimitaka@nihon-u.ac.jp (K.N.); katayama.daichi@nihon-u.ac.jp (D.K.); shimizu.shoichi@nihon-u.ac.jp (S.S.); matsuda.kengo@nihon-u.ac.jp (K.M.); tokunaga.wataru@nihon-u.ac.jp (W.T.); aoki.ryoji@nihon-u.ac.jp (R.A.); fuwa.kazumasa@nihon-u.ac.jp (K.F.); morioka.ichiro@nihon-u.ac.jp (I.M.)

**Keywords:** antioxidant effect, anti-inflammatory effect, glutathione, 1-methylnicotinamide, metabolite analyses

## Abstract

This study investigated the mechanism of reducing body fat via whey protein diet. Pregnant mice were fed whey or casein, and their offspring were fed by birth mothers. After weaning at 4 weeks, male pups received the diets administered to their birth mothers (n = 6 per group). At 12 weeks of age, body weight, fat mass, fasting blood glucose (FBG), insulin (IRI), homeostatic model assessment of insulin resistance (HOMA-IR), cholesterol (Cho), triglyceride (TG), the expression levels of lipid metabolism-related genes in liver tissues and metabolomic data of fat tissues were measured and compared between the groups. The birth weights of pups born were similar in the two groups. Compared to the pups in the casein group, at 12 weeks of age, pups in the whey group weighed less, had significantly lower fat mass, HOMA-IR and TG levels (*p* < 0.01, *p* = 0.02, *p* = 0.01, respectively), and significantly higher levels of the antioxidant glutathione and the anti-inflammatory 1-methylnicotinamide in fat tissues (*p* < 0.01, *p* = 0.04, respectively). No differences were observed in FBG, IRI, Cho levels (*p* = 0.75, *p* = 0.07, *p* = 0.63, respectively) and expression levels of lipid metabolism-related genes. Whey protein has more antioxidant and anti-inflammatory properties than casein protein, which may be its mechanism for reducing body fat.

## 1. Introduction

Japan is one of the developed countries where average birth weight has decreased and the birth rate of low-birth-weight (LBW) infants has not declined [1]. LBW infants have an elevated risk of developing diseases such as obesity and type 2 diabetes mellitus in adulthood. The fetus undergoes physiological changes to adapt to its environment when undernourished in utero, including slowed weight gain, resulting in relative overnutrition when the nutritional environment improves after birth. The developmental origins of health and disease (DOHaD) theory [2] affirm that disease risks need to be fully understood to avoid their development over the lifespan. For pediatricians, the DOHaD theory supports the idea that nutritional management in early childhood and pre-adolescence is necessary to prevent disease development in the first place [3]. This study focused on whey protein, a nutrient that can potentially protect LBW infants against metabolic syndrome later in life.

Whey protein, a nutrient-rich dairy protein that is abundant in dairy products such as yogurt and cheese, is associated with many health benefits. Whey protein is considered a functional food and has been increasingly demanded as a dietary supplement in recent years [4]. Whey protein is also present in breast milk and artificial formulas. Protein composition in breast milk changes over the lactation period. Colostrum consists of 90% whey protein and 10% casein protein; however, the ratio shifts to 60% whey and 40% casein in mature breast milk. In contrast, cow milk usually consists of 20% whey and 80% casein proteins [4]. Major components of whey protein include lactoferrin, beta-lactoglobulin, alpha-lactalbumin, glycomacropeptide, and immunoglobulin [5]. Increasing evidence demonstrates the health-promoting effects of whey protein at the biochemical level, including:

1 Glucose metabolism effect
  Whey protein improvement of insulin resistance by inhibiting the secretion of serotonin in peripheral tissues and fibroblast growth factor 21 in liver tissue [6].
2 Muscle protein synthesis effect
  Whey protein promotion of muscle synthesis by activating the mammalian target of rapamycin (mTOR), a metabolic pathway required for muscle synthesis [7].
3 Anti-inflammatory effect
  In a murine hepatitis model, whey protein suppression of the production of inflammatory cytokines, thereby inhibiting hepatocyte necrosis and apoptosis [8]. Similar results were found in clinics, where it suppressed the inflammatory response in COPD patients [9].
4 Antioxidant effect
  Whey protein exhibition of antioxidant activities in vitro [10].
5 Lipid metabolism effect
  Whey protein promotes triglyceride degradation and inhibits fatty acid synthesis in mice by affecting transcription factors involved in lipid metabolism [11].

Recently, clinical reports found LBW infants with non-obese type 2 diabetes mellitus. Kuwabara et al. reported that LBW infants raised with adequate nutrition after birth often develop type 2 diabetes in adulthood and that, at the time of onset, they had a significant accumulation of visceral fat compared to subcutaneous fat [12]. Nagano et al. determined that non-obese LBW infants in pre-adolescence often develop type 2 diabetes in adulthood, and their body fat is in the normal range, while their muscle mass is deficient [13], suggesting that muscle mass and lipid metabolism may be involved in the pathogenesis of type 2 diabetes mellitus in individuals born as LBW infants. As a cause of this, it has been reported that preterm infants have higher levels of oxidative stress markers compared to full-term infants [14]. Moreover, males are reported to have higher levels of oxidative stress markers than females; as a result, males are more prone to type 2 diabetes and cardiovascular events [15,16]. Due to the effects of whey protein in promoting muscle synthesis and improving glucose and lipid metabolisms, along with its anti-inflammatory and antioxidant activities, feeding LBW infants a diet rich in whey protein during infancy, early childhood, and pre-adolescence may help to prevent them from developing diabetes later in life. However, the mechanism by which whey protein exerts these effects remains to be perfectly elucidated [17].

Therefore, this study aimed to investigate the effect of whey protein on glucose and lipid metabolism and identify the potential mechanism involved in body fat reduction by measuring physical and biochemical changes in male mice exposed to whey protein from embryonic development to adulthood in comparison to mice raised exposed to casein protein diet over the same period.

## 2. Materials and Methods

### 2.1. Experimental Animals

All experimental protocols and procedures were approved by the Animal Experimentation Committee of Nihon University Itabashi Hospital (approval ID: AP20MED018-1, approval date: 5 June 2020). Pregnant Institute of Cancer Research (ICR) dams at gestational day 2 (GD2) were purchased from Sankyo Labo Service Corporation, Inc. (Tokyo, Japan).

### 2.2. Rearing Conditions

**Figure 1:**
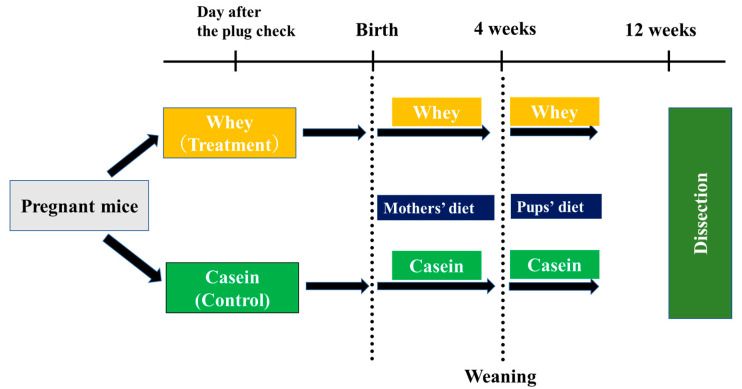
Experimental procedures. Study flow.

ICR pregnant mice were divided into two groups upon arrival, the casein and whey dietary groups. After birth, male pups were selected and raised on the same diet as their mothers. All mice were reared under the temperature of 22 ± 2 °C, humidity of 55 ± 5%, and 12/12 h light/dark cycles. In the casein group, mice were reared on AIN-93G, a standard rodent feed administered during pregnancy and growth periods in murine experiments (casein 20%, L-cystine 0.3%, corn starch 39.7486%, alpha-corn starch 13.2%, sucrose 10.0%, soybean oil 7.0%, cellulose powder 5.0%, mineral 3.5%, vitamin 1.0%, choline bitartrate 0.25%, tertiary butyl hydroquinone 0.0014%: energy 359 kcal) (Oriental Yeast Co., Ltd. Tokyo, Japan) [18]. In the whey group, the mice were reared on a modified blend of AIN-93G in which the casein component was replaced by whey. Pups were reared to 12 weeks of age before physical and biochemical measurements (Figure 1).

### 2.3. Body Weight

Pups were weighed once a week from birth to 12 weeks of age.

### 2.4. Blood Glucose, Serum Insulin, and Insulin Resistance (HOMA-IR)

At 12 weeks of age, adult male mice were fasted for 12 h and then dissected under isoflurane inhalation anesthesia (5% induction, 2% maintenance). Blood was collected from the heart by cardiac puncture via a midline incision. Blood glucose levels were measured using a Stat Strip XP2 (Nipro, Osaka, Japan). Next, serum was separated from total blood by centrifugation at 3000 rpm for 5 min and stored at −20 °C. Serum insulin was assessed for immunoreactive insulin levels (IRI) using a mouse/rat total insulin (high sensitivity) assay kit (Immuno-Biological Laboratories Co., Fujioka, Gunma, Japan). Serum was also assayed for insulin resistance using the human formula for the homeostasis model assessment of insulin resistance (HOMA-IR) [19].

### 2.5. Body Composition and Fat Weight

Body composition was measured using a bioelectrical impedance spectroscopy (BIS) device for laboratory animals (ImpediVET^TM^: Bioresearch Center, Co., Ltd., Nagoya, Japan) [20]. To estimate fat mass (FM) and fat-free mass (FFM), we measured the BIS differences in the electrical conductivity of biological tissues since adipose tissue is less conductive than muscle and other tissues due to lower water per unit volume. Fat weight was evaluated, and all observable adipose tissue was dissected.

### 2.6. Serum Lipoprotein Fractionation

Serum lipoproteins were separated into distinct fractions based on their cholesterol and triglyceride contents using gel-permeation high-performance liquid chromatography (HPLC) according to a method previously described (LipoSEARCH^®^; Skylight Biotech, Akita, Japan) [21,22,23]. Cholesterol and triglyceride values were estimated in total and for each of the major lipoprotein classes: very-low-density lipoprotein (VLDL), low-density lipoprotein (LDL), and high-density lipoprotein (HDL) based on the peaks in the HPLC elution profile corresponding to different lipoprotein particle sizes [22].

### 2.7. Gene Expression Analysis of Liver Tissue

RNA expression levels of the genes related to lipid metabolism in the liver (*PPARα*, *PPARγ*, *SREBP1c*, *HSL*, and *LPL*) were measured using real-time quantitative polymerase chain reaction (RT-qPCR). RNA was isolated from frozen liver tissue of male mice (n = 5 per group) using the protocol provided by ReliaPrep RNA Miniprep Systems (Promega Corporation, Madison, WI, USA). RNA was reverse-transcribed to complementary DNA using ReverTra Ace qPCR RT Master Mix (Toyobo Co., Ltd., Osaka, Japan) on an ABI Geneamp 9700 PCR-Thermal Cycler (Applied Biosystems, Thermo Fisher Scientific Inc., Tokyo, Japan). RT-qPCR was performed using KOD-Plus-Ver.2 polymerase mix (Toyobo Co., Ltd.) on an ABI Applied Biosystems 7300 Real-Time PCR System (Applied Biosystems, Thermo Fisher Scientific Inc.). In this study, we used the same primers as in a previous report [11] as a reference. These primers were manufactured by Takara Bio Inc. (Kusatsu, Japan).

### 2.8. Metabolomic Analysis of Adipose Tissue

A sample of frozen adipose tissue from male mice (approximately 50 mg, n = 5 per group) was placed in a homogenization tube with zirconia beads (5-mmφ and 3-mmφ), to which 1500 µL of 50% acetonitrile/Milli-Q water containing internal standards (H3304-1002, Human Metabolome Technologies, Inc. Yamagata, Japan) was added. Two cycles of homogenization at 1500 rpm for 120 s at 4 °C were performed using a beaded shaker (Shake Master NEO, BioMedical Science, Tokyo, Japan). Next, the sample was centrifuged at 2300× *g* for 5 min at 4 °C to remove high-molecular-weight components. Then, 400 μL supernatant was collected, centrifuged at 9100× *g* for 120 min at 4 °C, and filtered using a Millipore 5-kDa cut-off filter (Human Metabolome Technologies, Inc. (HMT), Tsuruoka, Yamagata, Japan). Finally, the filtrate was dried by vacuum evaporation and dissolved in 50 µL Milli-Q water. This solution was subjected to metabolomic analysis using capillary electrophoresis time-of-flight mass spectrometry [24,25] on an Agilent CE system (Agilent Technologies, Inc., Santa Clara, CA, USA). Peak area, *m*/*z*, and migration time data of the mass spectrum peaks (range: 50–1000 *m*/*z*) were calculated for peaks automatically detected using integrated software (Keio University, Shizuoka, Japan) [26]. The chemical species associated with each peak was identified based on its *m*/*z* value and migration time with reference to the HMT metabolite database. Relative levels of each metabolite were calculated by normalizing the peak area with the internal standards and sample volume.

Principal component analysis and hierarchical cluster analysis were performed according to previously described methods [27]. 

### 2.9. Serum and Urine Creatinine

Serum samples were collected as described in Section 2.4, and serum creatinine was measured using enzymatic method. Urine creatinine was measured in 24 h urine samples, collected while mice were kept in a metabolic cage for laboratory animals, using a conventional creatinine deaminase-based enzymatic method.

### 2.10. Statistical Analysis

Data are reported as mean ± standard error of the mean. Each outcome was compared between the experimental (whey) and control (casein) groups using Mann–Whitney U test, using JMP statistical software (ver. 14.0: SAS Institute, Cary, NC, USA). When *p* < 0.05, the differences were considered statistically significant, and when 0.05 < *p* < 0.10, the differences were considered marginally significant.

## 3. Results

### 3.1. Body Weight History

**Figure 2:**
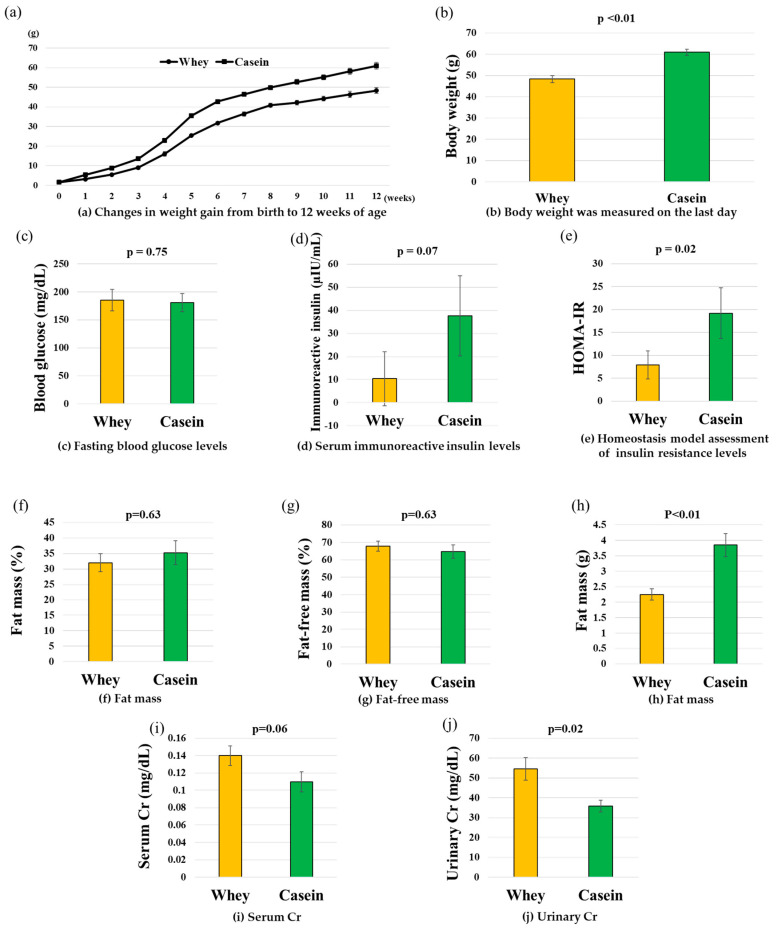
Body weight and glucose metabolism markers. (**a**) Changes in weight gain from birth to 12 weeks of age (●: Whey, ■: Casein). (**b**) Body weight was measured on the last day. (**c**) Fasting blood glucose levels. (**d**) Serum immunoreactive insulin levels. (**e**) Homeostasis model assessment of insulin resistance levels. (**f**) Fat mass (%). (**g**) Fat-free mass (%). (**h**) Fat mass (g). (**i**) Serum Cr. (**j**) Urinary Cr. Data are shown as the mean ± standard error of the mean (n = 6 per group).

Body weight at birth was not significantly different between the two groups. However, every week thereafter, the weight was lower in the whey group than in the casein group. At 12 weeks, body weight was significantly lower in the whey group than in the casein group (48.3 g vs. 61.0 g, *p* < 0.01) (Figure 2a,b).

### 3.2. Blood Glucose, IRI, and HOMA-IR

Fasting blood glucose levels were not significantly different between the two groups (177.5 mg/dL vs. 184.7 mg/dL, *p* = 0.75). IRI was marginally lower in the whey than in the casein group (22.0 μIU/mL vs. 47.0 μIU/mL, *p* = 0.07). HOMA-IR was significantly lower in the whey than in the casein group (7.9 vs. 19.2, *p* = 0.02) (Figure 2c–e).

### 3.3. Fat Weight and Body Composition

Fat weight was significantly lower in the whey than in the casein group (2.4 g vs. 3.8 g, *p* < 0.01). However, body composition was similar in both groups in terms of FFM (67.9% vs. 64.7%, *p* = 0.63) and FM (32.0% vs. 35.3 %, *p* = 0.63) (Figure 2f–h).

### 3.4. Serum and Urine Creatinine

Creatinine levels were marginally higher in serum in the whey group than in the casein group (0.11 mg/dL vs. 0.14 mg/dL, *p* = 0.06) and significantly higher in urine (35.8 mg/dL vs. 54.6 mg/dL, *p* = 0.02) (Figure 2i,j).

### 3.5. Serum Lipoprotein Fractions

**Figure 3:**
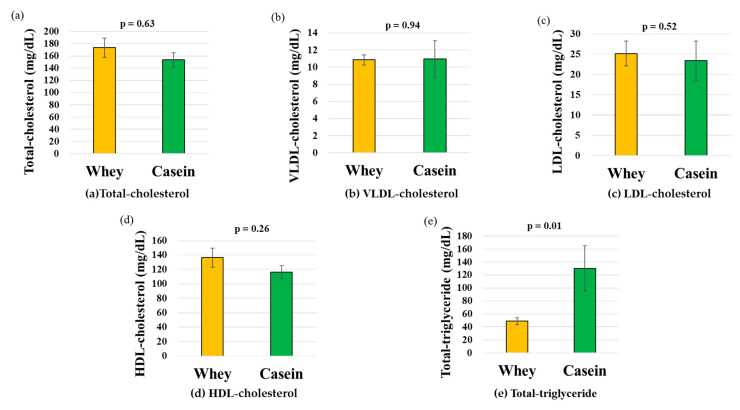
Body composition and serum lipoprotein levels. (**a**) Total cholesterol. (**b**) VLDL, (**c**) LDL, and (**d**) HDL-cholesterol. (**e**) Total triglyceride. Data are shown as the mean ± standard error of the mean (n = 6 per group). HDL, high-density lipoprotein; LDL, low-density lipoprotein; VLDL, very-low-density lipoprotein.

For cholesterol levels, no significant differences in total or individual values were observed between the two groups (total: 173.51 mg/dL vs. 153.46 mg/dL, *p* = 0.63; VLDL: 10.85 mg/dL vs. 10.94 mg/dL, *p* = 0.94; LDL: 25.16 mg/dL vs. 23.38 mg/dL, *p* = 0.52; HDL: 136.44 mg/dL vs. 116.16 mg/dL, *p* = 0.26) (Figure 3a–d). In contrast, triglyceride levels were significantly lower in the whey group than in the casein group for every outcome measured (total: 51.47 mg/dL vs. 119.2 mg/dL, *p* = 0.01) (Figure 3e).

### 3.6. Hepatic Gene Expression

**Figure 4:**
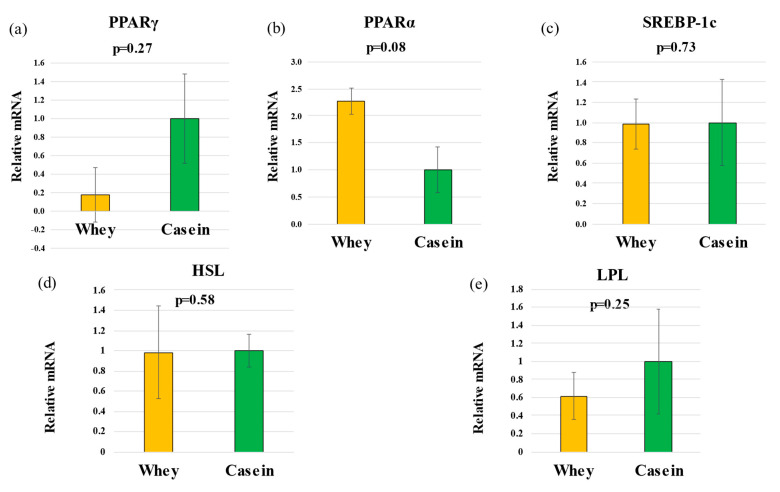
Relative mRNA levels. (**a**) PPARγ, (**b**) PPARα, (**c**) SREBP-1c, (**d**) HLS, and (**e**) LPL. (n = 5 per group). PPARγ, peroxisome proliferator-activated receptor γ; PPARα, peroxisome proliferator-activated receptor α; SREBP-1c, sterol regulatory element-binding protein-1c; HSL, hormone-sensitive lipase; LPL, lipoprotein lipase.

RT-qPCR analysis showed that the hepatic expression of *PPARα* was marginally higher in the whey than in the casein group (*p* = 0.08); however, no other differences were observed for any of the other lipid metabolism-related genes evaluated (*PPARγ*, *p* = 0.27; *SREBP1c*, *p* = 0.73; *HSL*, *p* = 0.58; *LPL*: *p* = 0.25) (Figure 4a–e).

### 3.7. Adipose Metabolism

**Figure 5:**
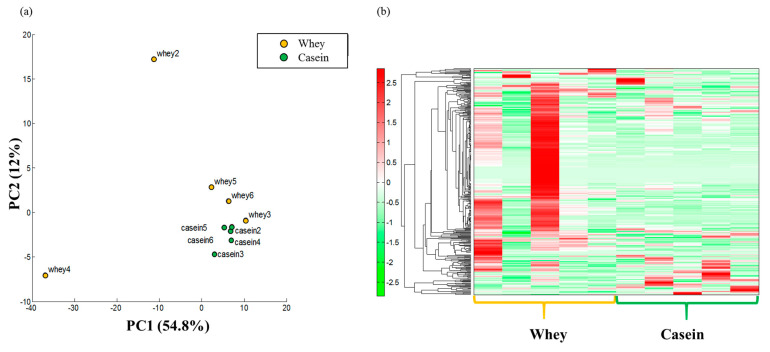
Metabolite analyses in fatty tissue. (**a**) Principal component (PC) analysis. (**b**) Heat map display of the hierarchical cluster analysis (n = 5 per group).

**Figure 6:**
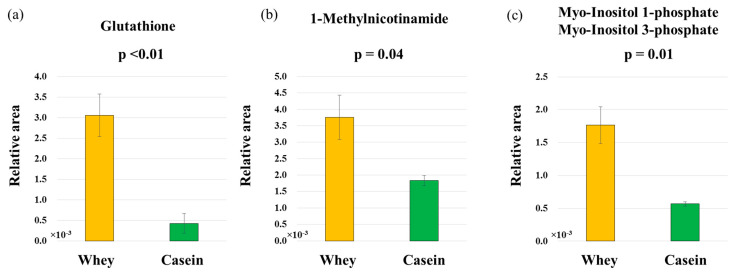
Comprehensive comparative analysis between the whey and casein groups. (**a**) Glutathione. (**b**) 1-Methylnicotinamide. (**c**) Myo-Inositol 1-phosphate and Myo-Inositol 3-phosphate (n = 5 per group).

Results of the main component analysis or the hierarchical clustering heatmap did not show clear differences between the groups (Figure 5a,b; Supplementary Tables S1–S3). Table 1 shows the metabolites that were measured and associated with antioxidant and anti-inflammatory effects. The levels of glutathione, 1-methylnicotinamide, and myo-inositol phosphates (1-phosphate + 3-phosphate) were significantly higher in the whey group than in the casein group (*p* < 0.01, *p* = 0.04, and *p* = 0.01) (Figure 6a–c).

## 4. Discussion

**Figure 7:**
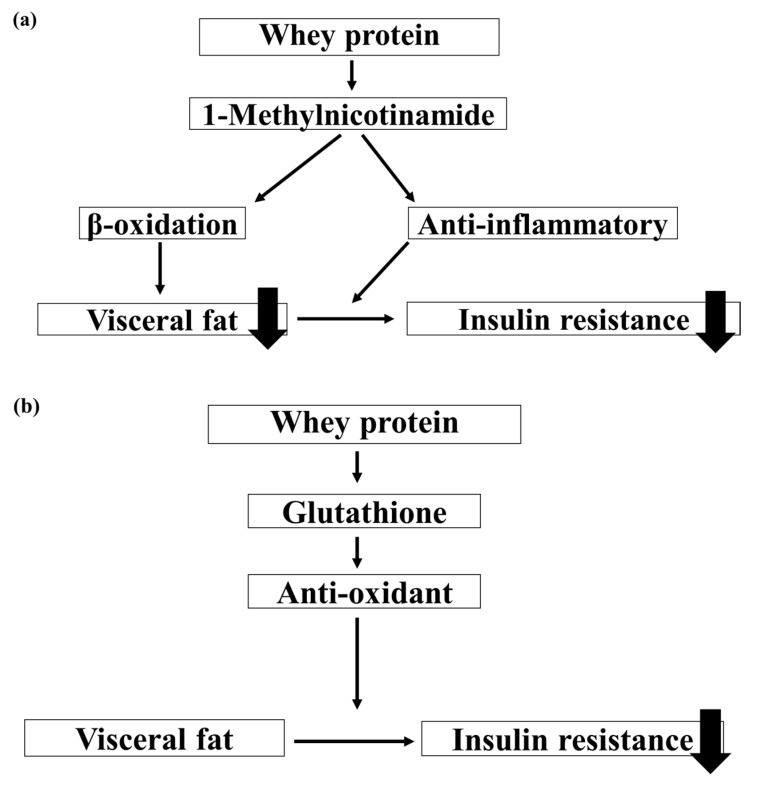
Schematical representation of the theory for the mechanism by which whey protein ameliorates lipid and glucose metabolisms. (**a**) 1-Methylnicotinamide and (**b**) Glutathione.

In the present study, whey protein intake activated lipid metabolism, reduced fat mass, and decreased insulin resistance in the mouse model. We theorize that these results were obtained because whey protein intake accelerated β-oxidation and anti-inflammatory and antioxidant activities (Figure 7a,b).

### 4.1. Lipid Metabolism

We found that mice raised on a whey protein diet had significantly lower level serum total-triglyceride than the ones raised on a casein protein diet. In addition, mice in the whey protein group had significantly higher *PPARα* RNA expression that those in the casein group. A previous report showed that whey protein increases *PPARα* expression [11], and that *PPARα* increases intracellular mitochondrial β-oxidation and activates lipid metabolism [28]. Therefore, we speculate that whey protein intake promotes triglyceride utilization by increasing *PPARα* expression. Furthermore, the adipose tissue of these mice in the whey protein group contained significantly higher levels of 1-methylnicotinamide, a metabolite of nicotinamide, compared to the casein group. Since 1-methylnicotinamide has anti-inflammatory and β-oxidation-limiting effects [29], we hypothesize that whey protein intake improves lipid metabolism by regulating β-oxidation (Figure 7a).

### 4.2. Glucose Metabolism

In adipocytes, disrupting the mechanisms regulating adipocytokine production results in the excessive production of inflammatory cytokines, leading to insulin resistance [30]. In addition, severe oxidative stress decreases and inactivates insulin receptors of adipocytes, resulting in reduced gene expression and secretion of insulin in these cells [31,32]. Our metabolomic analysis indicated that levels of 1-methylnicotinamide and glutathione in adipose tissue were significantly higher in the whey group than in the casein group. We speculate that increased 1-methylnicotinamide due to whey protein intake suppressed chronic inflammation in adipocytes, thereby improving insulin resistance (Figure 7a). Whey protein also exerts antioxidant effects by increasing glutathione levels [33,34]; therefore, this is another plausible mechanism for the amelioration of insulin resistance observed (Figure 7b).

### 4.3. Improvement Myogenic Insulin Resistance

Compared to soy protein, whey protein has been reported to decrease the circulating levels of interleukin-6 and tumor necrosis factor-α and affect muscle metabolism [35]. In our study, whey protein was found to increase the level of the anti-inflammatory marker 1-methylnicotinamide in adipose tissue compared to casein protein. Greater muscle mass due to elevated serum creatine can reduce myogenic insulin resistance [36]. In this study, we did not measure muscle mass, and the two groups analyzed had statistically similar body compositions. From these results, whey protein may have the potential to reduce visceral fat more than subcutaneous fat. One possible reason why no difference was observed in FFM could be due to the relatively short duration of the intervention period for both whey and casein protein diets. However, we determined that serum creatine was significantly higher in the whey group than in the casein group. Whey protein is commonly used as a dietary supplement to increase muscle mass [37] along with increasing creatine; thus, whey protein may have influenced creatinine in this study. Elevated levels of serum creatine caused by whey protein intake may improve insulin resistance.

### 4.4. Myo-Inositol Phosphates

In the present study, metabolome analysis showed that myo-inositol 1-phosphate and myo-inositol 3-phosphate levels in adipose tissue were significantly higher in the whey group than in the casein group. Myo-inositol is a component of membrane phospholipids that plays a role in signal transduction. Rats with compromised myo-inositol expression show high liver triglyceride content [38]. Both myo-inositol 1-phosphate and myo-inositol 3-phosphate belong to the myo-inositol metabolic pathway. Myo-inositol improves insulin resistance [39]. Although no reports of improved glucose or lipid metabolism directly caused by myo-inositol 1-phosphate or myo-inositol 3-phosphate are available, our findings suggest a potential connection.

### 4.5. Infant and Oxidative Stress

Matsubasa et al. collected urine samples from fifty Japanese very-low-birthweight infants on various days after birth and measured the oxidative stress marker, 8-hydroxydeoxyguanosine. Their results showed that urine 8-hydroxydeoxyguanosines in very-low-birthweight infants were higher than those in full-term infants, and that oxidative stress marker levels decreased as the weight increased after birth [40]. Piyush et al. also reported that small-for-gestational age infants born to malnourished mothers had higher levels of the oxidative stress marker, malondialdehyde, and lower levels of enzymes in the antioxidative systems, such as superoxide dismutase, catalase, and glutathione peroxidase than appropriate-for-gestational age infants to healthy mothers [41]. These results suggest that infants with low birth weight and high prenatal stress had higher oxidative stress and lower antioxidant capacity. In our current study, it was found that nutrition with whey protein from the neonatal period improved antioxidant and anti-inflammatory capacity. Therefore, it may be possible to feed these children with whey protein to reduce oxidative stress and improve antioxidant capacity.

### 4.6. Comparison of the Antioxidant and Anti-Inflammatory Effects of Breast Milk and Formula

Breast milk is considered the best source of nutrition for infants in many respects. Breast milk contains carbohydrates, proteins, fats, vitamins, minerals, digestive enzymes and hormones. The protein composition of breast milk adapts to the growth of the child, which changes over time, and the proportion of whey protein and casein protein changes [42]. Breast milk is also considered superior to artificial milk in terms of antioxidant and anti-inflammatory properties. Aycicek et al. examined fifty-four healthy-term infants fed breast milk or a cow’s milk modified formula and found that oxidative stress markers were lower in the breast milk group [43]. In a study using a human intestinal model, Allan et al. determined that breast milk reduced interleukin-8, a marker of inflammation in the intestinal epithelium, down-regulated toll-like receptor 4 expression, and suppressed inflammatory responses [44]. These reports suggested that the superior antioxidant and anti-inflammatory effects of breast milk compared to formula are due to the higher proportion of whey protein in breast milk. Therefore, changing the protein ratio and increasing the proportion of whey protein over casein protein may strengthen the antioxidant and anti-inflammatory effects. Oxidative stress is a contributing factor to cell damage and the exacerbation of several chronic diseases. Dietary antioxidants aid in fighting against free radicals and thereby prevent or reduce oxidative stress. Corrochano et al. reported that oxidative stress contributes to cell injury and aggravates several chronic diseases, and compared whey from different milk sources and contextualized whey proteins within the broader spectrum of known food antioxidants [45]. However, for whey proteins to be effective in boosting antioxidant levels in target organs, their antioxidant activity must survive not only processing, but also upper gut transit and arrival in the bloodstream. In this study, it was shown that direct cell exposure to whey samples can increase intracellular antioxidants such as glutathione. The physiological relevance of these in vitro assays is questionable, and there is conflicting evidence from dietary intervention trials involving rats and humans that whey products can boost cellular antioxidant biomarkers.

## 5. Future Directions

We will continue to test whey protein interventions in an LBW, non-obese, hyperglycemic mouse model and obese animal models with high-fat diet challenge to examine its effects on fat weight and insulin resistance. Furthermore, since mice were reared exclusively on either whey protein or casein protein in these experiments, examining mixed interventions in which whey and casein are administered together at different ratios will be necessary. Such mixed formulations must be investigated to apply the interventions in clinical practice.

## 6. Conclusions

Whey protein intervention started in the fetal period seems to increase the levels of several metabolites with anti-inflammatory and antioxidant effects, leading to reduced fat weight and improved insulin resistance.

## Figures and Tables

**Table 1:** Summary of metabolite analysis in the adipose tissue.

**(a) Antioxidant Markers**			
		Comparative Analysis	
		Group Whey vs. Casein	
Category	Compound name	Ratio	*p*-value
Antioxidant	Ascorbic acid	1.1	0.775
	Carnosine	22	0.323
	Glutathione	7.1	0.004
	Hypotaurine	29	0.286
	Tartaric acid	0.6	0.458
**(b) Anti-inflammatory markers**			
		Comparative Analysis	
		Group Whey vs. Casein	
Category	Compound name	Ratio	*p*-value
Anti-inflammatory	1-Methylnicotinamide	2.0	0.044
	Histidine	1.6	0.243
**(c) Glucose metabolism markers**			
		Comparative Analysis	
		Group Whey vs. Casein	
Category	Compound name	Ratio	*p*-value
Glucose metabolism	myo-Inositol phosphates	3.1	0.013

## Data Availability

The data that support the findings of this study are available from the corresponding author upon reasonable request.

## Supplementary Materials

The following supporting information can be downloaded at: https://www.mdpi.com/article/10.3390/nu15102263/s1. Table S1: principal component score; Table S2: metabolites and principal component score; Table S3: Results of comparative analysis.
