# Supplementary material for: Body Fat-Reducing Effects of Whey Protein Diet in Male Mice
Source: Nutrients. 2023 May 10;15(10):2263. doi: 10.3390/nu15102263 (PMC10223508; doi:10.3390/nu15102263)
Supplement: Supplementary file 1 [file nutrients-15-02263-s001.zip › Supplementary Table S1.pdf]

Supplementary Table S1. Principal component score

	Contribution rate (%)	Whey					Casein				
		whey2	whey3	whey4	whey5	whey6	casein2	casein3	casein4	casein5	casein6
**PC1**	54.74	-11.31	10.39	-36.93	2.29	6.28	7.13	3.04	7.02	5.30	6.79
**PC2**	11.92	17.19	-0.89	-7.06	2.83	1.27	-1.63	-4.71	-3.16	-1.71	-2.12
**PC3**	9.84	-3.20	9.10	2.46	4.77	6.72	0.03	-7.20	-1.95	-10.28	-0.46
**PC4**	5.86	1.19	-0.03	-0.10	-1.21	-5.56	11.52	0.65	0.39	-5.03	-1.82
**PC5**	4.49	-0.23	4.11	1.14	-1.94	-3.33	1.86	-8.04	-1.41	6.76	1.07
**PC6**	4.03	0.06	-4.35	-0.01	-0.59	0.10	0.62	-2.66	-0.67	-2.54	10.04
**PC7**	3.58	-2.00	-5.54	0.07	3.28	5.19	4.12	-2.10	-3.32	2.87	-2.56
**PC8**	3.26	0.36	-2.56	0.25	-2.13	2.06	0.04	-3.97	8.58	-0.72	-1.91
**PC9**	2.27	-1.03	-1.12	-0.24	6.97	-4.27	-1.21	-1.06	1.98	0.08	-0.08

PC, principal component
