# Supplementary material for: Body Fat-Reducing Effects of Whey Protein Diet in Male Mice
Source: Nutrients. 2023 May 10;15(10):2263. doi: 10.3390/nu15102263 (PMC10223508; doi:10.3390/nu15102263)
Supplement: Supplementary file 1 [file nutrients-15-02263-s001.zip › Supplementary Table S2.pdf]

Supplementary Table S2. Metabolites and principal component score

	Compound name	PubChem CID	HMDB ID	*m/z*	MT/RT	PC1	PC2
A_0003	Crotonic acid	637090		85.029	9.15	1.7E-01	4.0E-01
A_0005	Butyric acid<br>Isobutyric acid	264<br>6590	HMDB0000039<br>HMDB0001873	87.045	8.43	-8.2E-01	-2.8E-02
A_0006	Lactic acid	612	HMDB0000190,HMDB0001311	89.024	9.14	-9.7E-01	-2.3E-01
A_0007	Isovaleric acid<br>DL-2-Methylbutyric Acid<br>Valeric acid	10430<br>8314<br>7991	HMDB0000718<br>HMDB0002176<br>HMDB0000892	101.061	7.95	-8.6E-01	3.0E-01
A_0008	3-Hydroxybutyric acid	441	HMDB0000011,HMDB0000357,HMDB0000442	103.040	8.16	-2.4E-01	-1.4E-01
A_0009	2-Hydroxybutyric acid	440864	HMDB0000008	103.040	8.36	-2.6E-01	-3.6E-01
A_0010	2-Hydroxyisobutyric acid	11671	HMDB0000729	103.040	8.45	-9.7E-01	-1.3E-01
A_0011	Glyceric acid	439194	HMDB0000139,HMDB0006372	105.019	8.81	-7.7E-01	-4.7E-01
A_0012	Fumaric acid	444972	HMDB0000134	115.003	17.83	-8.9E-01	1.6E-01
A_0014	Hexanoic acid	8892	HMDB0000535	115.076	7.66	-6.8E-01	1.5E-01
A_0015	*N*-Acetylglycine	10972	HMDB0000532	116.035	8.14	-7.4E-01	4.1E-01
A_0016	Succinic acid	1110	HMDB0000254	117.019	15.74	-9.1E-01	-3.2E-01
A_0017	β-Hydroxyisovaleric acid	69362	HMDB0000754	117.055	7.84	-5.5E-01	1.3E-01
A_0018	2-Hydroxyvaleric acid	98009	HMDB0001863	117.056	7.94	-9.7E-01	1.4E-01
A_0019	Isethionic acid	7866	HMDB0003903	124.991	9.70	-9.8E-01	-1.0E-01
A_0020	5-Oxoproline	7405	HMDB0000267	128.035	8.10	-5.4E-01	1.8E-01
A_0021	5-Oxohexanoic acid	18407		129.056	7.79	1.7E-01	-1.1E-01
A_0022	4-Methyl-2-oxovaleric acid<br>3-Methyl-2-oxovaleric acid<br>2-Oxohexanoic acid	70<br>47<br>159664	HMDB0000695<br>HMDB0000491<br>HMDB0001864	129.056	8.18	-7.0E-01	-3.8E-01
A_0023	Heptanoic acid	8094	HMDB0000666	129.092	7.41	-4.1E-01	1.3E-01
A_0024	*N*-Acetylalanine	88064	HMDB0000766	130.051	7.61	1.5E-01	5.4E-01
A_0025	6-Hydroxyhexanoic acid	14490		131.071	7.29	-9.1E-01	3.6E-01
A_0026	2-Hydroxy-4-methylvaleric acid	439960	HMDB0000624	131.072	7.59	-9.5E-01	2.0E-01
A_0027	Malic acid	525	HMDB0000156,HMDB0000744	133.014	15.97	-8.4E-01	2.8E-01
A_0028	Threonic acid	5460407	HMDB0000943	135.030	7.90	-8.8E-01	-2.9E-01
A_0029	6-Hydroxynicotinic acid	72924	HMDB0002658	138.019	8.04	3.3E-01	-2.2E-01
A_0030	Ethanolamine phosphate	1015	HMDB0000224	140.012	6.89	-9.9E-01	-1.1E-01
A_0031	*N*-Ethylmaleimide_+H_2O	4362		142.051	7.47	-1.2E-02	3.5E-01
A_0032	Octanoic acid	379	HMDB0000482	143.107	7.20	-5.1E-01	6.3E-01
A_0033	XA0004			144.031	7.97	-9.3E-01	3.1E-01
A_0034	4-Acetamidobutanoic acid	18189	HMDB0003681	144.066	7.39	-2.8E-01	2.2E-01
A_0035	2-Oxoglutaric acid	51	HMDB0000208	145.013	15.92	-9.1E-01	-3.7E-01
A_0036	2-Hydroxyglutaric acid	43	HMDB0000606,HMDB0000694	147.029	13.51	-7.4E-01	-3.4E-01
A_0037	Tartaric acid	444305	HMDB0000956	149.009	16.39	-2.7E-01	-2.3E-01
A_0038	3-Phenylpropionic acid	107	HMDB0000764	149.060	7.56	-1.4E-01	7.3E-01
A_0039	Cysteinesulfinic acid	1549098	HMDB0000996	152.003	8.15	-9.4E-02	6.1E-01
A_0040	Orotic acid	967	HMDB0000226	155.010	8.41	-9.7E-01	-1.7E-01
A_0041	Dihydroorotic acid	439216	HMDB0000528	157.025	8.00	-9.1E-01	-3.7E-01
A_0042	2-Oxooctanoic acid	67600		157.087	7.33	1.7E-01	2.8E-02
A_0043	Pelargonic acid	8158	HMDB0000847	157.123	7.02	-6.4E-01	1.8E-01
A_0044	8-Hydroxyoctanoic acid	69820		159.103	6.94	4.3E-01	-6.0E-02
A_0045	2-Hydroxyoctanoic acid	94180	HMDB0000711	159.103	7.01	-8.2E-01	-3.1E-01
A_0046	3-Hydroxy-3-methylglutaric acid	1662		161.045	12.60	-2.8E-01	9.1E-01
A_0047	*N*-Acetylcysteine	12035	HMDB0001890	162.024	7.54	-2.0E-01	6.5E-01
A_0048	*o*-Coumaric acid<br>*p*-Coumaric acid	637540<br>637542	HMDB0002641<br>HMDB0002035	163.039	7.46	1.8E-01	-8.6E-02
A_0049	4-Hydroxyphenylglyoxylic acid	355		165.019	11.08	-9.1E-01	-3.7E-01
A_0050	Terephthalic acid	7489	HMDB0002428	165.019	13.29	7.1E-01	-1.3E-01
A_0051	Perillic acid	1256	HMDB0004586	165.091	7.02	-4.0E-01	-2.6E-01
A_0052	XA0012			166.018	8.02	-9.4E-01	-9.3E-02
A_0053	Phosphoenolpyruvic acid	1005	HMDB0000263	166.974	15.83	-5.4E-01	5.9E-01
A_0054	Uric acid	1175	HMDB0000289	167.021	7.62	-7.1E-01	-3.7E-02
A_0055	Homogentisic acid<br>*p*-Hydroxymandelic acid	780<br>328	HMDB0000130<br>HMDB0000822	167.035	7.38	-9.7E-01	-2.0E-01
A_0056	Dihydroxyacetone phosphate	668	HMDB0001473	168.990	10.61	-9.8E-01	6.9E-02
A_0058	Glycerol 2-phosphate	2526		171.006	10.44	-9.1E-01	-3.7E-01
A_0059	Glycerol 3-phosphate	439162	HMDB0000126	171.006	10.16	-9.5E-01	3.1E-01
A_0060	Decanoic acid	2969	HMDB0000511	171.139	6.87	-3.7E-01	3.7E-01
A_0061	Isovalerylalanine<br>*N*-Acetylleucine	129285<br>70912	HMDB0000747<br>HMDB0011756	172.098	6.92	-2.3E-01	3.2E-01
A_0062	*cis*-Aconitic acid	643757	HMDB0000072	173.009	18.72	-9.3E-01	-2.1E-01
A_0063	Suberic acid	10457	HMDB0000893	173.082	10.74	5.3E-01	-2.4E-01
A_0064	*N*-Acetylaspartic acid	65065	HMDB0000812	174.041	11.95	-6.2E-01	-3.1E-01
A_0065	Ascorbic acid	54670067	HMDB0000044	175.024	7.28	-2.2E-01	1.6E-01

A_0066	*N*-Carbamoylaspartic acid	93072	HMDB0000828	175.035	12.50	-9.1E-01	-3.7E-01
A_0067	Homovanillic acid<br>Hydroxyphenyllactic acid	1738<br>9378	HMDB0000118<br>HMDB0000755	181.050	7.11	-9.6E-01	8.5E-02
A_0068	Homocysteic acid	177491	HMDB0002205	182.013	8.02	2.6E-01	-4.7E-02
A_0069	*O*-Phosphoserine	68841	HMDB0000272	184.001	10.30	-9.7E-02	6.6E-01
A_0070	2-Phosphoglyceric acid	439278	HMDB0003391	184.985	14.76	-5.6E-01	5.1E-01
A_0071	3-Phosphoglyceric acid	439183	HMDB0000807	184.986	15.04	-5.4E-01	5.9E-01
A_0072	Undecanoic acid	8180	HMDB0000947	185.154	6.73	-1.5E-01	1.3E-01
A_0073	XA0017			186.114	6.85	-4.6E-01	3.6E-01
A_0074	*N*-Acetylglutamine	25561	HMDB0006029	187.073	6.96	-9.1E-01	-3.7E-01
A_0075	Azelaic acid	2266	HMDB0000784	187.098	10.23	3.7E-01	-1.3E-01
A_0076	10-Hydroxydecanoic acid	74300		187.134	6.67	-8.0E-01	-4.2E-01
A_0077	Kynurenic acid	3845	HMDB0000715	188.036	7.35	-9.7E-01	-1.6E-01
A_0078	*N*-Acetylglutamic acid	70914	HMDB0001138	188.056	10.99	-8.3E-01	-2.0E-01
A_0079	*N*-Acetylmethionine	448580	HMDB0011745	190.054	6.99	-5.7E-01	4.6E-01
A_0080	Isocitric acid	1198	HMDB0000193	191.019	19.08	-3.6E-01	-5.0E-01
A_0081	Citric acid	311	HMDB0000094	191.020	18.16	-9.7E-01	-1.8E-01
A_0082	XA0019			191.020	7.16	-9.8E-01	-2.1E-02
A_0083	Quinic acid	6508	HMDB0003072	191.056	6.97	1.8E-01	2.3E-01
A_0084	Phenaceturic acid	68144	HMDB0000821	192.066	7.06	-5.3E-01	2.2E-01
A_0085	Galacturonic acid<br>Glucuronic acid	439215<br>94715	HMDB0002545<br>HMDB0000127	193.035	6.97	-5.0E-01	1.0E-01
A_0086	Gluconic acid	10690	HMDB0000625	195.051	7.05	-9.3E-01	1.7E-02
A_0088	Lauric acid	3893	HMDB0000638	199.171	6.62	-4.3E-01	4.1E-01
A_0089	Sebacic acid	5192	HMDB0000792	201.112	9.81	1.7E-01	-1.1E-01
A_0090	Xanthurenic acid	5699	HMDB0000881	204.029	10.29	1.3E-01	-9.0E-02
A_0091	Indole-3-lactic acid<br>5-Methoxyindoleacetic acid	676157<br>18986	HMDB0000671<br>HMDB0004096	204.066	7.06	-9.8E-01	4.7E-02
A_0092	Mucic acid	3037582	HMDB0000639	209.030	11.60	-9.6E-01	-1.2E-01
A_0093	Phosphocreatine	9548602	HMDB0001511	210.028	10.26	-1.6E-01	-2.8E-01
A_0094	3-Indoxylsulfuric acid	10258	HMDB0000682	212.002	8.14	6.5E-02	1.2E-01
A_0095	Tridecanoic acid	12530	HMDB0000910	213.185	6.50	-6.8E-01	2.4E-01
A_0096	Pantothenic acid	6613	HMDB0000210	218.103	6.61	-8.3E-01	3.3E-01
A_0097	Myristoleic acid	5281119	HMDB0002000	225.186	6.46	-4.4E-01	2.5E-01
A_0098	Myristic acid	11005	HMDB0000806	227.201	6.44	-8.1E-01	9.0E-02
A_0099	Ribulose 5-phosphate	439184	HMDB0000618	229.011	9.29	-5.9E-01	7.0E-01
A_0100	Ribose 5-phosphate	439167	HMDB0001548	229.011	8.95	-2.2E-01	8.9E-01
A_0101	XA0033			242.080	6.57	-9.4E-01	3.3E-01
A_0102	XA0080	440992		243.027	8.65	-9.0E-01	-3.7E-01
A_0103	γ-Glu-Taurine	68759	HMDB0004195	253.051	6.98	-9.7E-01	1.9E-01
A_0104	Ascorbate 2-sulfate	54676864		254.982	11.79	-6.8E-01	5.8E-02
A_0105	XA0035			254.982	11.28	-4.7E-02	1.1E-01
A_0106	Glucose 1-phosphate	65533	HMDB0001586	259.022	8.62	-9.2E-01	3.4E-01
A_0107	Fructose 6-phosphate	603	HMDB0000124	259.022	8.48	-8.5E-01	4.9E-01
A_0108	*myo*-Inositol 1-phosphate<br>*myo*-Inositol 3-phosphate	107737<br>440194	HMDB0000213<br>HMDB0006814	259.022	8.75	-4.4E-01	3.1E-01
A_0109	*myo*-Inositol 2-phosphate	160886		259.022	8.94	-4.8E-02	8.4E-01
A_0110	Glucose 6-phosphate	5958	HMDB0001401	259.023	8.39	-7.1E-01	6.7E-01
A_0111	2,3-Diphosphoglyceric acid	186004	HMDB0001294	264.950	14.41	-2.2E-02	1.9E-01
A_0112	6-Phosphogluconic acid	91493	HMDB0001316	275.016	12.02	-5.8E-01	7.6E-01
A_0113	Xanthosine	64959	HMDB0000299	283.066	6.56	-7.6E-01	6.1E-01
A_0114	Orotidine	92751	HMDB0000788	287.051	6.80	-9.7E-01	-1.8E-01
A_0115	Sedoheptulose 7-phosphate	165007	HMDB0001068	289.032	8.21	-5.5E-01	7.4E-01
A_0116	Retinoic acid	444795	HMDB0001852	299.202	6.24	-7.7E-02	7.2E-01
A_0117	*N*-Acetylglucosamine 1-phosphate	440272	HMDB0001367	300.048	8.16	-9.3E-01	3.2E-01
A_0118	*N*-Acetylglucosamine 6-phosphate	440996	HMDB0001062	300.048	7.84	-6.5E-01	6.5E-01
A_0119	cCMP<br>2',3'-cCMP	19236<br>68934	 HMDB0011691	304.035	6.56	7.5E-02	-2.5E-01
A_0120	*N*-Acetylneuraminic acid	439197	HMDB0000230	308.099	6.22	-9.9E-01	-1.5E-02
A_0121	Ribulose 1,5-diphosphate	123658		308.980	12.65	-3.3E-01	7.6E-01
A_0123	3'-CMP	66535		322.044	8.34	1.7E-01	-2.9E-01
A_0124	CMP	6131	HMDB0000095	322.044	8.17	-7.9E-01	-5.5E-01
A_0125	UMP	6030	HMDB0000288	323.026	8.33	-5.9E-01	-7.0E-01
A_0126	*N*-Glycolylneuraminic acid	440001	HMDB0000833	324.093	6.20	2.3E-01	-2.3E-01
A_0127	cAMP	6076	HMDB0000058	328.045	6.46	-9.1E-01	-3.7E-01
A_0128	Fructose 1,6-diphosphate	172313	HMDB0001058	338.990	11.93	-9.8E-01	9.3E-02
A_0130	AMP	6083	HMDB0000045	346.054	7.91	-7.0E-01	-6.0E-01
A_0131	3'-AMP	41211	HMDB0003540	346.054	8.26	1.6E-01	5.8E-03
A_0132	IMP	8582	HMDB0000175	347.038	8.12	-8.1E-01	-1.6E-01
A_0133	Prostaglandin E_2	5280360	HMDB0001220	351.219	6.02	-8.8E-01	4.6E-01
A_0134	Prostaglandin $F_{2\alpha}$	5280363	HMDB0001139	353.234	5.98	-8.2E-01	5.5E-01

A_0135	GMP	6804	HMDB0001397	362.049	7.81	-5.0E-01	-6.9E-01
A_0136	XA0055			368.999	11.74	-8.0E-01	5.0E-01
A_0137	NADPH_divalent	5884	HMDB0000221	371.539	9.49	-9.8E-01	-1.6E-02
A_0138	CoA_divalent	87642	HMDB0001423	382.548	8.98	1.1E-01	1.1E-01
A_0139	PRPP	7339	HMDB0000280	388.945	13.19	-9.1E-01	-3.7E-01
A_0140	FAD_divalent	643975	HMDB0001248	391.570	6.77	-9.7E-01	-8.6E-02
A_0142	CDP	6132	HMDB0001546	402.012	9.61	-9.1E-01	-3.7E-01
A_0143	UDP	6031	HMDB0000295	402.994	9.75	-9.4E-01	-3.2E-01
A_0144	Acetyl CoA_divalent	444493	HMDB0001206	403.552	8.70	-9.1E-01	-3.7E-01
A_0145	Cholic acid	221493	HMDB0000619	407.280	5.95	3.2E-01	-8.3E-02
A_0146	Thiamine diphosphate	1132	HMDB0001372	423.031	6.83	-8.8E-01	-4.4E-01
A_0148	ADP	6022	HMDB0001341	426.022	9.16	-9.3E-01	-3.2E-01
A_0149	3',5'-ADP	159296	HMDB0000061	426.022	11.02	-4.0E-01	1.4E-01
A_0150	GDP	8977	HMDB0001201	442.017	8.96	-9.5E-01	-2.3E-01
A_0151	XA0065			445.053	6.01	-9.8E-01	-6.8E-02
A_0152	Adenylosuccinic acid	447145	HMDB0000536	462.068	11.34	-2.9E-01	-3.0E-01
A_0155	CTP	6176	HMDB0000082	481.979	10.27	-9.1E-01	-3.7E-01
A_0156	UTP	6133	HMDB0000285	482.961	10.43	-9.3E-01	-3.3E-01
A_0157	CDP-choline	13804	HMDB0001413	487.100	5.88	-9.3E-01	-9.2E-02
A_0159	ATP	5957	HMDB0000538	505.990	9.82	-9.2E-01	-3.4E-01
A_0160	Taurocholic acid	46783527	HMDB0000036	514.287	5.83	2.0E-01	5.1E-02
A_0161	GTP	6830	HMDB0001273	521.988	9.60	-9.6E-01	-2.4E-01
A_0162	ADP-ribose	445794	HMDB0001178	558.064	7.28	2.5E-01	-4.1E-01
A_0163	UDP-galactose<br>UDP-glucose	23724458<br>8629	HMDB0000302<br>HMDB0000286	565.053	7.39	-9.8E-01	-3.0E-03
A_0164	UDP-glucuronic acid	17473	HMDB0000935	579.024	9.30	-9.0E-01	-1.5E-01
A_0165	GDP-fucose<br>ADP-glucose	10918995<br>16500	HMDB0001095<br>HMDB0006557	588.079	7.14	-6.9E-01	-4.0E-01
A_0166	NAD^+	5893	HMDB0000902	662.099	5.67	-9.3E-01	-3.3E-01
A_0167	$NADP^+$	5886	HMDB0000217	742.064	7.98	-1.0E+00	4.4E-02
C_0001	Urea	1176	HMDB0000294	61.040	18.03	-9.3E-01	-8.7E-02
C_0002	Ethanolamine	700	HMDB0000149	62.060	5.19	-9.1E-01	-1.9E-03
C_0003	3-Aminopropionitrile	1647	HMDB0004101	71.060	5.21	2.6E-01	-4.7E-02
C_0004	XC0001			72.081	5.22	-2.8E-01	9.1E-01
C_0005	Aminoacetone	215	HMDB0002134	74.060	5.61	-9.1E-01	-3.7E-01
C_0006	Gly	750	HMDB0000123	76.039	6.85	-9.8E-01	2.0E-01
C_0007	Trimethylamine *N*-oxide	1145	HMDB0000925	76.076	5.40	-9.6E-01	-2.5E-01
C_0008	Morpholine	8083	HMDB0031581	88.076	5.43	-7.2E-01	2.3E-01
C_0009	Putrescine	1045	HMDB0001414	89.107	3.84	-9.3E-01	2.6E-01
C_0010	β-Ala	239	HMDB0000056	90.055	6.01	-9.7E-01	-1.9E-01
C_0011	Ala	602	HMDB0000161,HMDB0001310	90.055	7.42	-7.4E-01	-8.2E-02
C_0012	Sarcosine	1088	HMDB0000271	90.055	7.81	-9.4E-01	-2.8E-01
C_0013	Dimethylaminoethanol	7902	HMDB0032231	90.091	5.66	-8.8E-01	-2.4E-01
C_0014	Glycerol	753	HMDB0000131	93.055	18.85	2.6E-01	2.9E-01
C_0015	Phenol	996	HMDB0000228	95.047	4.41	-9.2E-01	-1.9E-01
C_0016	Cyclohexylamine	7965		100.112	6.26	-5.6E-01	2.6E-02
C_0017	Acetoacetamide	80077		102.055	18.87	1.8E-01	-1.2E-01
C_0018	Homoserinelactone	73509		102.056	5.78	-9.1E-01	-3.7E-01
C_0019	Cadaverine	273	HMDB0002322	103.123	4.06	-6.6E-01	5.4E-01
C_0020	*N*,*N*-Dimethylglycine	673	HMDB0000092	104.071	9.03	-7.8E-01	-4.6E-02
C_0021	GABA	119	HMDB0000112	104.071	6.30	-9.2E-01	-3.6E-01
C_0022	2-Aminoisobutyric acid<br>2-Aminobutyric acid	6119<br>6657	HMDB0001906<br>HMDB0000452	104.071	7.91	-6.2E-02	-4.5E-01
C_0023	3-Aminobutyric acid	10932		104.071	6.50	2.0E-01	-1.6E-01
C_0024	Choline	305	HMDB0000097	105.110	5.61	-9.8E-01	1.4E-01
C_0025	Ser	617	HMDB0000187,HMDB0003406	106.050	8.26	-9.8E-01	1.5E-01
C_0026	Diethanolamine	8113	HMDB0004437	106.086	6.25	1.3E-01	9.2E-02
C_0027	Hypotaurine	107812	HMDB0000965	110.027	14.99	-9.6E-01	-2.3E-01
C_0029	Histamine	774	HMDB0000870	112.086	3.90	-8.6E-01	-7.6E-03
C_0030	Uracil	1174	HMDB0000300	113.034	18.86	-5.1E-01	7.7E-01
C_0031	Creatinine	588	HMDB0000562	114.066	5.97	-7.3E-01	-1.8E-01
C_0032	3-Amino-2-piperidone	5200225	HMDB0000323	115.086	6.21	1.7E-02	2.5E-02
C_0033	Pro	614	HMDB0000162,HMDB0003411	116.070	8.90	-9.8E-01	-5.9E-02
C_0034	Guanidoacetic acid	763	HMDB0000128	118.061	6.75	2.2E-02	-3.6E-01
C_0035	Val	1182	HMDB0000883	118.086	8.21	-9.7E-01	1.5E-01
C_0036	Betaine	247	HMDB0000043	118.086	9.29	-9.9E-01	-4.4E-02
C_0037	5-Aminovaleric acid	138	HMDB0003355	118.086	6.58	-3.9E-01	5.3E-01
C_0038	Thr	6288	HMDB0000167	120.065	8.68	-9.9E-01	-7.1E-02
C_0039	Homoserine	12647	HMDB0000719	120.065	8.31	-9.0E-01	8.1E-02
C_0040	Betaine aldehyde_+H_2O	249	HMDB0001252	120.102	6.09	-4.2E-01	6.4E-01
C_0041	4-Hydroxyphenethyl alcohol_-H_2O	10393	HMDB0004284	121.065	18.91	-6.3E-01	-1.6E-01

C_0042	Anserine_divalent	112072	HMDB0000194	121.069	5.57	-9.5E-01	-2.5E-01
C_0043	Cys	594	HMDB0000574,HMDB0003417	122.028	9.35	-6.3E-01	2.8E-01
C_0044	2-Amino-2-(hydroxymethyl)-1,3-propanediol	6503		122.081	6.77	1.3E-01	-9.0E-02
C_0045	Nicotinamide	936	HMDB0001406	123.055	6.08	-8.5E-01	4.2E-01
C_0046	Nicotinic acid	938	HMDB0001488	124.039	8.27	1.8E-01	-8.6E-02
C_0047	Taurine	1123	HMDB0000251	126.022	18.84	-9.8E-01	-8.7E-02
C_0048	1-Methylhistamine	3614	HMDB0000898	126.103	4.01	-5.1E-01	8.2E-01
C_0049	3-Hydroxy-2-methyl-4-pyrone	8369	HMDB0030776	127.038	18.92	1.1E-01	-5.9E-01
C_0051	Imidazole-4-acetic acid	96215	HMDB0002024	127.050	6.57	-4.0E-01	8.3E-01
C_0052	XC0016			129.066	7.17	-7.8E-01	-1.8E-01
C_0053	4-Oxopyrrolidine-2-carboxylic acid	107541		130.050	9.05	-9.6E-01	-8.2E-03
C_0054	Pipecolic acid	439227	HMDB0000070,HMDB0000716,HMDB0005960	130.087	8.43	-9.3E-01	-3.4E-01
C_0055	*trans* -Glutaconic acid	5280498	HMDB0000620	131.034	19.62	-8.3E-01	-3.2E-01
C_0056	*N* -Acetylputrescine	122356	HMDB0002064	131.118	6.90	-1.5E-01	4.9E-01
C_0057	Hydroxyproline	5810	HMDB0000725	132.066	9.94	-9.8E-01	-1.2E-01
C_0058	3-Guanidinopropionic acid	67701		132.076	6.56	-8.1E-01	-2.1E-01
C_0059	6-Aminohexanoic acid	564	HMDB0001901	132.102	6.77	-9.8E-01	1.3E-01
C_0060	Leu	857	HMDB0000687	132.102	8.47	-9.5E-01	3.0E-01
C_0061	Ile	791	HMDB0000172	132.102	8.37	-9.5E-01	2.7E-01
C_0062	Gly-Gly	11163	HMDB0011733	133.060	6.85	-4.4E-01	-2.9E-02
C_0063	Asn	236	HMDB0000168,HMDB0033780	133.060	8.69	-9.9E-01	4.1E-03
C_0064	Creatine	586	HMDB0000064	133.079	7.24	-9.7E-01	-1.6E-01
C_0065	Ornithine	389	HMDB0000214,HMDB0003374	133.097	5.57	-4.6E-01	5.1E-01
C_0066	Thiaproline	9934		134.027	11.62	-6.1E-01	1.1E-01
C_0067	Asp	424	HMDB0000191,HMDB0006483	134.044	9.60	-7.4E-01	-1.9E-01
C_0068	Adenine	190	HMDB0000034	136.062	6.24	-9.6E-01	2.2E-02
C_0069	Hypoxanthine	790	HMDB0000157	137.046	9.42	-6.1E-01	7.6E-01
C_0070	1-Methylnicotinamide	457	HMDB0000699	137.071	6.02	-9.2E-01	1.5E-01
C_0072	Trigonelline	5570	HMDB0000875	138.056	8.62	-8.8E-01	4.4E-01
C_0073	Tyramine	5610	HMDB0000306	138.091	6.80	-6.9E-01	-1.8E-01
C_0074	γ-Glu-Lys_divalent	65254	HMDB0029154	138.582	7.02	-7.2E-01	-3.3E-01
C_0075	Urocanic acid	736715	HMDB0000301	139.050	6.79	1.0E-01	-7.1E-02
C_0076	1*H* -Imidazole-4-propionic acid	10105257		141.066	6.64	1.2E-02	-4.4E-02
C_0077	1-Methyl-4-imidazoleacetic acid	75810	HMDB0002820	141.066	6.79	9.6E-02	-3.0E-02
C_0078	XC0029<br>Stachydrine	0<br>115244	 HMDB0004827	144.101	9.44	-8.1E-01	-1.3E-01
C_0079	4-Guanidinobutyric acid	500	HMDB0003464	146.092	6.79	-5.2E-01	-8.8E-02
C_0080	γ-Butyrobetaine	134	HMDB0001161	146.118	6.63	-9.6E-01	-2.3E-01
C_0081	Acetylcholine	187	HMDB0000895	146.118	6.22	-9.7E-01	-1.9E-01
C_0082	Spermidine	1102	HMDB0001257	146.165	3.70	-5.8E-01	9.0E-02
C_0083	Lys	866	HMDB0000182,HMDB0003405	147.113	5.62	-9.8E-01	-1.6E-01
C_0084	2-Methylthiazolidine-4-carboxylic acid	160736		148.042	11.83	-6.8E-01	7.0E-01
C_0085	Isoglutamic acid	73064		148.060	7.52	5.7E-01	-5.8E-02
C_0086	*N* -Acetylserine	65249	HMDB0002931	148.060	19.78	-6.8E-01	3.5E-02
C_0087	*threo* -β-Methylaspartic acid	440064		148.060	10.22	-7.6E-02	6.0E-01
C_0088	*N* -Methylaspartic acid	22880	HMDB0002393	148.060	11.34	-9.1E-01	-3.7E-01
C_0089	Gln	738	HMDB0000641,HMDB0003423	148.079	8.88	-9.8E-01	-1.4E-01
C_0090	Glu	611	HMDB0000148,HMDB0003339	149.063	9.04	-9.5E-01	-1.0E-01
C_0091	Met	876	HMDB0000696	150.058	8.84	-9.6E-01	7.3E-02
C_0092	Triethanolamine	7618		150.112	6.74	2.5E-01	-3.5E-01
C_0093	Guanine	764	HMDB0000132	152.057	6.83	-4.4E-01	-2.8E-01
C_0094	γ-Glu-Arg_divalent	20719180	HMDB0029143	152.585	7.13	-6.5E-01	-3.2E-01
C_0095	Xanthine	1188	HMDB0000292	153.041	16.64	-5.9E-01	7.7E-01
C_0096	N^1-Methyl-4-pyridone-5-carboxamide	440810	HMDB0004194	153.066	15.59	-9.3E-01	8.4E-02
C_0097	Octopamine<br>Dopamine	440266<br>681	HMDB0004825<br>HMDB0000073	154.087	7.11	-9.5E-01	2.6E-01
C_0098	His	773	HMDB0000177	156.077	5.98	-9.9E-01	-3.0E-02
C_0099	Imidazolelactic acid	793		157.061	7.22	-3.8E-01	4.9E-01
C_0100	XC0145<br>Ala-Ala	15331<br>5460362	 HMDB0003459	161.091	7.57	-1.2E-01	-4.0E-01
C_0101	N^6-Methyllysine	164795	HMDB0002038	161.128	5.80	-9.4E-01	-2.4E-01
C_0102	*O* -Acetylhomoserine<br>2-Aminoadipic acid	439389<br>92136	 HMDB0000510	162.076	9.04	-6.4E-01	-3.3E-01
C_0103	5-Hydroxylysine	3032849	HMDB0000450	163.107	5.84	-5.9E-01	-7.6E-01
C_0104	Carnitine	85	HMDB0000062	163.116	6.95	-9.6E-01	-2.2E-01
C_0105	Lumazine	10250		165.041	18.77	2.1E-01	4.4E-01
C_0106	Methionine sulfoxide	158980	HMDB0002005	166.053	9.84	-3.6E-01	-8.5E-01
C_0107	7-Methylguanine	11361	HMDB0000897	166.072	6.75	-8.7E-01	3.4E-01
C_0108	Normetanephrine_-H_2O	688100	HMDB0000819	166.086	7.48	-4.7E-01	7.6E-01
C_0109	Phe	994	HMDB0000159	166.086	9.12	-9.3E-01	3.5E-01
C_0110	Taurocyamine	68340	HMDB0003584	168.042	18.85	6.9E-01	-3.1E-01

C_0111	Pyridoxal	1050	HMDB0001545	168.065	7.18	-2.0E-01	-1.4E-01
C_0112	4-Hydroxyphenylglycine<br>3-Methoxyanthranilic acid	92143<br>255720		168.065	9.97	1.5E-01	6.7E-02
C_0113	Tyr-Arg_divalent	123804		169.594	6.29	1.1E-01	-4.6E-01
C_0114	Noradrenaline<br>6-Hydroxydopamine	439260<br>4624	HMDB0000216<br>HMDB0001537	170.082	7.38	-9.3E-01	3.3E-01
C_0115	1-Methylhistidine<br>3-Methylhistidine	92105<br>64969	HMDB0000001<br>HMDB0000479	170.092	6.13	-7.4E-01	-5.4E-01
C_0116	XC0040			174.087	9.93	-8.9E-01	4.0E-01
C_0117	*N*-Acetylornithine	439232	HMDB0003357	175.108	7.80	9.0E-02	-3.5E-01
C_0118	N^5-Ethylglutamine	439378		175.109	9.07	-9.0E-01	-1.0E-01
C_0119	Arg	6322	HMDB0000517,HMDB0003416	175.119	5.82	-9.5E-01	6.4E-02
C_0120	Guanidinosuccinic acid	439918	HMDB0003157	176.067	8.32	-8.3E-01	2.3E-02
C_0121	Citrulline	9750	HMDB0000904	176.103	9.12	-9.1E-01	-2.7E-01
C_0122	Serotonin	5202	HMDB0000259	177.103	7.17	-7.5E-01	-3.3E-01
C_0123	Alliin	87310		178.053	12.55	1.7E-01	-1.7E-01
C_0124	Gluconolactone	7027	HMDB0000150	179.055	19.61	-9.0E-01	-4.8E-02
C_0125	Glucosamine	439213	HMDB0001514	180.089	7.58	-6.3E-01	-3.5E-01
C_0126	Tyr	1153	HMDB0000158	182.081	9.36	-9.7E-01	1.4E-01
C_0127	Phosphorylcholine	1014	HMDB0001565	184.074	17.28	-9.7E-01	2.2E-01
C_0128	Adrenaline	5816	HMDB0000068	184.097	7.49	-2.8E-01	9.1E-01
C_0129	N^1-Acetylspermidine	496	HMDB0001276	188.176	5.15	-9.8E-01	-2.0E-02
C_0130	*N*-Acetyllysine	92907	HMDB0000446	189.123	8.01	-4.9E-01	-2.4E-01
C_0131	Gly-Leu			189.123	7.90	1.2E-01	-2.6E-01
C_0132	N^6-Acetyllysine	92832	HMDB0000206	189.123	9.42	-5.8E-01	-1.2E-01
C_0133	N_{ω}-Methylarginine	132862		189.134	6.09	-8.9E-01	4.1E-01
C_0134	N^6,N^6,N^6-Trimethyllysine	440120	HMDB0001325	189.160	5.86	-8.9E-01	-2.5E-01
C_0135	Homocitrulline	65072	HMDB0000679	190.118	9.20	-8.1E-01	-2.8E-01
C_0136	Gly-Asp	97363		191.067	8.12	-9.9E-01	-3.1E-02
C_0137	4-Aminohippuric acid	2148	HMDB0001867	195.078	8.38	-2.8E-01	9.1E-01
C_0138	Tyrosine methyl ester	70652		196.097	7.50	1.7E-01	-1.1E-01
C_0139	*N*-Acetylhistidine	75619		198.087	8.04	-6.2E-01	-2.9E-02
C_0140	ADMA	123831	HMDB0001539	203.150	6.26	-9.5E-01	1.3E-01
C_0141	SDMA	169148	HMDB0003334	203.150	6.36	-9.1E-01	-3.6E-02
C_0142	Spermine	1103	HMDB0001256	203.223	3.65	-2.4E-01	8.1E-02
C_0143	*O*-Acetylcarnitine	439756	HMDB0000201	204.122	7.40	-9.9E-01	-8.2E-02
C_0144	γ-Glu-Gly	165527	HMDB0011667	205.082	9.96	-7.8E-01	5.8E-01
C_0145	Trp	1148	HMDB0000929	205.097	9.07	-8.6E-01	-9.0E-02
C_0146	Carboxymethyllysine	123800		205.119	7.58	-9.7E-01	-1.0E-01
C_0147	Lipoamide	863	HMDB0000962	206.069	19.18	-2.8E-01	9.1E-01
C_0148	Kynurenine	846	HMDB0000684	209.092	8.27	-9.6E-01	-2.4E-01
C_0149	Propionylcarnitine<br>XC0061	188824<br>0	HMDB0000824	218.138	7.63	-8.3E-01	-8.1E-02
C_0150	β-Ala-Lys	440638		218.150	5.53	-1.8E-01	-3.6E-01
C_0151	γ-Glu-Ala	440103	HMDB0006248	219.098	10.18	-7.4E-01	2.5E-01
C_0152	XC0065			221.091	10.84	-9.7E-01	-1.8E-01
C_0153	*N*-Acetylglucosylamine	439454	HMDB0001104	221.112	8.12	-5.4E-01	2.7E-01
C_0154	*N*-Acetylgalactosamine<br>*N*-Acetylglucosamine<br>*N*-Acetylmannosamine	35717<br>439174<br>439281	HMDB0000853<br>HMDB0000215<br>HMDB0001129	222.097	18.87	-6.4E-01	-1.5E-01
C_0155	Cystathionine	834	HMDB0000099	223.075	8.16	-9.5E-01	-2.7E-01
C_0156	Neostigmine	4456		223.145	7.29	7.5E-02	-2.5E-01
C_0157	3-Hydroxykynurenine	11811	HMDB0011631	225.085	8.16	-9.1E-01	-3.7E-01
C_0158	Carnosine	439224	HMDB0000033	227.114	5.51	-9.4E-01	-2.9E-01
C_0159	2'-Deoxycytidine	13711	HMDB0000014	228.098	7.73	-4.5E-01	-1.9E-01
C_0160	Butyrylcarnitine	439829	HMDB0002013	232.154	7.83	-9.0E-01	-3.0E-01
C_0161	Isobutyrylcarnitine	168379	HMDB0000736	232.154	7.77	-4.0E-01	-1.6E-01
C_0162	γ-Glu-Ser	22844748	HMDB0029158	235.093	10.42	-9.2E-01	2.5E-01
C_0163	Thr-Asp	3280446		235.093	8.73	-9.2E-01	-3.9E-02
C_0164	Ser-Glu			235.093	8.54	-9.8E-01	-1.0E-01
C_0165	*N'*-Formylkynurenine	910	HMDB0001200	237.087	10.05	-9.1E-01	-3.7E-01
C_0166	Cystine	595	HMDB0000192	241.032	9.09	4.2E-01	-2.8E-01
C_0167	Homocarnosine	10243361	HMDB0000745	241.130	5.57	-9.6E-01	-2.2E-01
C_0168	Thymidine	5789	HMDB0000273	243.098	18.85	-2.1E-01	-2.6E-01
C_0169	Cytidine	6175	HMDB0000089	244.093	7.93	-8.3E-01	5.3E-01
C_0170	Uridine	6029	HMDB0000296	245.077	18.89	-7.8E-01	5.8E-01
C_0171	N^1-Acetylspermine	916	HMDB0001186	245.233	4.51	-9.1E-01	-3.7E-01
C_0172	Isovalerylcarnitine	6426851	HMDB0000688	246.170	8.00	-7.0E-01	9.0E-02
C_0173	γ-Glu-Val	7015683	HMDB0011172	247.129	10.51	-9.7E-01	-7.9E-02
C_0174	Malonylcarnitine	22833583	HMDB0002095	248.112	8.37	-9.2E-01	-1.8E-01
C_0175	Pyridoxamine 5'-phosphate	1053	HMDB0001555	249.063	8.61	-9.5E-01	4.8E-02

C_0176	γ-Glu-Thr	53861142	HMDB0029159	249.108	10.52	-9.5E-01	-2.3E-01
C_0177	γ-Glu-Cys	123938	HMDB0001049	251.069	10.60	-7.9E-01	1.5E-01
C_0178	2'-Deoxyinosine	135398593	HMDB0000071	253.094	15.41	1.8E-01	-8.6E-02
C_0179	XC0089			255.098	7.80	-9.9E-01	-3.3E-02
C_0180	XC0154	3182		255.107	18.93	5.1E-01	-4.8E-01
C_0181	Glycerophosphocholine	439285	HMDB0000086	258.108	18.43	-9.9E-01	-3.0E-02
C_0182	γ-Glu-Ile<br>γ-Glu-Leu	22885096<br>151023	HMDB0011170<br>HMDB0011171	261.143	10.68	-9.6E-01	2.5E-03
C_0183	γ-Glu-Asn	131801686	HMDB0029144	262.103	10.56	-8.9E-01	4.1E-01
C_0184	γ-Glu-Ornitine	189156	HMDB0002248	262.140	6.95	-4.7E-01	-3.4E-02
C_0185	γ-Glu-Asp	161197	HMDB0030419	263.088	10.76	-9.1E-01	-6.9E-02
C_0186	Thiamine	1130	HMDB0000235	265.111	5.35	-9.5E-01	2.7E-01
C_0187	Adenosine	60961	HMDB0000050	268.103	8.11	-9.3E-01	-3.3E-01
C_0188	Inosine	6021	HMDB0000195	269.088	16.60	-8.7E-01	4.4E-01
C_0189	γ-Glu-Gln	150914	HMDB0011738	276.119	10.77	-9.7E-01	1.2E-01
C_0190	Glu-Glu	439500		277.104	8.96	-6.6E-01	-4.7E-02
C_0191	γ-Glu-Glu	92865	HMDB0011737	277.103	10.86	-6.9E-01	6.1E-01
C_0192	Saccharopine	160556	HMDB0000279	277.139	8.84	-9.4E-01	-2.9E-01
C_0193	γ-Glu-Met	7009567	HMDB0034367	279.101	10.73	-9.1E-01	-3.7E-01
C_0194	1-Methyladenosine	27476	HMDB0003331	282.117	8.17	-9.7E-01	1.7E-01
C_0195	Guanosine	6802	HMDB0000133	284.099	10.51	-9.6E-01	2.2E-01
C_0196	γ-Glu-His	7017195	HMDB0029151	285.120	7.13	-9.8E-01	1.2E-01
C_0197	Octanoylcarnitine	11953814	HMDB0000791	288.217	8.52	-9.5E-01	-1.6E-01
C_0198	Ophthalmic acid	7018721	HMDB0005765	290.134	10.95	-1.5E-01	-1.6E-01
C_0199	Argininosuccinic acid	16950	HMDB0000052	291.130	7.75	-7.8E-01	3.7E-01
C_0200	γ-Glu-Phe	111299	HMDB0000594	295.130	10.78	-8.4E-01	-7.7E-02
C_0201	5'-Deoxy-5'-methylthioadenosine	439176	HMDB0001173	298.097	8.28	-9.4E-01	-3.1E-01
C_0202	N^1-Methylguanosine	96373	HMDB0001563	298.115	9.97	-9.4E-01	2.7E-01
C_0203	Arg-Glu			304.161	6.12	-3.1E-01	1.1E-01
C_0204	Glutathione (GSSG)_divalent	65359	HMDB0003337	307.084	10.07	-9.9E-01	-1.1E-01
C_0205	Glutathione (GSH)	124886	HMDB0000125	308.092	10.97	-6.9E-01	2.6E-01
C_0206	XC0126			310.116	12.48	-6.6E-01	-4.7E-01
C_0207	Tyr-Glu			311.122	9.16	-3.2E-01	2.4E-01
C_0208	γ-Glu-Tyr	94340	HMDB0011741	311.123	10.95	-7.5E-02	5.2E-01
C_0209	*S*-Methylglutathione	115260		322.107	11.08	-9.8E-01	9.4E-02
C_0210	XC0132			325.161	7.10	-9.6E-01	-1.6E-01
C_0211	NMN	14180	HMDB0000229	335.065	17.41	-8.9E-01	-3.9E-01
C_0212	Lauroylcarnitine	168381	HMDB0002250	344.280	9.14	-9.2E-01	-2.3E-01
C_0213	Thiamine phosphate	1131	HMDB0002666	345.078	8.78	-9.8E-01	1.5E-02
C_0214	XC0137			350.099	11.19	-9.4E-01	-1.5E-01
C_0215	Decarboxylated *S*-Adenosylmethionine	439415	HMDB0000988	355.157	4.58	1.7E-01	-1.7E-01
C_0216	Riboflavin	493570	HMDB0000244	377.147	18.76	-9.2E-01	2.6E-01
C_0217	*S*-Lactoylglutathione	440018	HMDB0001066	380.114	11.45	-9.1E-01	-3.7E-01
C_0218	*S*-Adenosylhomocysteine	439155	HMDB0000939	385.129	7.17	-7.0E-01	7.0E-01
C_0219	*S*-Adenosylmethionine	34755	HMDB0001185	399.144	5.83	-9.7E-01	-2.0E-01
C_0220	Cysteine glutathione disulfide	10455148	HMDB0000656	427.098	9.63	-3.9E-01	2.3E-01

MT, migration time; PC, principal component; RT, retention time
