# Supplementary material for: Body Fat-Reducing Effects of Whey Protein Diet in Male Mice
Source: Nutrients. 2023 May 10;15(10):2263. doi: 10.3390/nu15102263 (PMC10223508; doi:10.3390/nu15102263)
Supplement: Supplementary file 1 [file nutrients-15-02263-s001.zip › Supplementary Table S3.pdf]

Supplementary Table S3. Results of comparative analysis

ID	Metabolite	PubChem CID	HMDB ID	Concentration (nmol/g)														Comparative Analysis		
				treatment					Control					treatment		Control		treatment vs Control		
				whey2	whey3	whey4	whey5	whey6	casein2	casein3	casein4	casein5	casein6	Mean	S.D.	Mean	S.D.	Ratio [¶]	*p* -value [‖]	
A_0009	2-Hydroxybutyric acid	440864	HMDB0000008	0.6	N.D.	1.8	1.1	N.D.	1.2	2.8	N.D.	2.1	1.6	1.2	0.6	1.9	0.7	0.6	0.2	
A_0035	2-Oxoglutaric acid	51	HMDB0000208	N.D.	N.D.	4.5	N.D.	N.D.	N.D.	N.D.	N.D.	N.D.	N.D.	4.5	N.A.	N.A.	N.A.	1<	N.A.	
A_0013	2-Oxoisovaleric acid	49	HMDB0000019	N.D.	N.D.	N.D.	N.D.	N.D.	N.D.	N.D.	N.D.	N.D.	N.D.	N.A.	N.A.	N.A.	N.A.	N.A.	N.A.	
A_0070	2-Phosphoglyceric acid	439278	HMDB0003391	4.2	N.D.	2.5	2.6	1.0	N.D.	2.6	1.9	0.8	N.D.	2.6	1.3	1.8	0.9	1.5	0.4	
A_0008	3-Hydroxybutyric acid	441	HMDB0000011,HMDB0000357,HMDB0000442	37	15	51	31	26	23	49	44	95	12	32	14	44	32	0.7	0.5	
A_0071	3-Phosphoglyceric acid	439183	HMDB0000807	29	4.5	17	16	6.0	14	16	13	5.4	7.5	14	9.8	11	4.5	1.3	0.5	
A_0112	6-Phosphogluconic acid	91493	HMDB0001316	8.7	1.5	4.1	2.4	1.3	N.D.	N.D.	0.4	1.4	2.6	3.6	3.1	1.5	1.1	2.4	0.2	
A_0144	Acetyl CoA_divalent	444493		N.D.	N.D.	0.2	N.D.	N.D.	N.D.	N.D.	N.D.	N.D.	N.D.	0.2	N.A.	N.A.	N.A.	1<	N.A.	
C_0068	Adenine	190	HMDB0000034	0.3	0.08	0.7	0.2	0.2	0.06	0.10	0.09	0.07	0.08	0.3	0.2	0.08	0.02	4.1	0.1	
C_0187	Adenosine	60961	HMDB0000050	6.4	0.5	109	3.4	3.5	1.3	2.2	2.0	1.2	0.7	25	47	1.5	0.6	16.6	0.3	
A_0148	ADP	6022	HMDB0001341	12	2.1	152	5.9	3.8	4.9	5.4	3.1	3.6	2.8	35	66	3.9	1.1	9.0	0.3	
C_0011	Ala	602	HMDB0000161,HMDB0001310	110	75	133	98	81	89	110	92	87	120	99	23	99	14	1.0	1.0	
A_0130	AMP	6083	HMDB0000045	32	26	170	34	67	70	110	74	62	47	66	60	73	23	0.9	0.8	
C_0071	Anthranilic acid	227	HMDB0001123	N.D.	N.D.	N.D.	N.D.	N.D.	N.D.	N.D.	N.D.	N.D.	N.D.	N.A.	N.A.	N.A.	N.A.	N.A.	N.A.	
C_0119	Arg	6322	HMDB0000517,HMDB0003416	23	6.1	34	9.6	15	12	13	11	13	13	17	11	13	0.9	1.4	0.4	
C_0063	Asn	236	HMDB0000168,HMDB0033780	16	5.9	25	10	9.2	8.4	11	9.6	9.7	10	13	7.3	9.7	0.8	1.4	0.3	
C_0067	Asp	424	HMDB0000191,HMDB0006483	44	26	61	31	24	27	55	32	44	35	37	16	39	11	1.0	0.9	
A_0159	ATP	5957	HMDB0000538	6.6	0.3	188	1.5	0.5	0.7	0.8	0.3	0.4	0.2	39	83	0.5	0.2	82.2	0.4	
C_0036	Betaine	247	HMDB0000043	48	9.6	117	21	9.9	13	12	10	7.9	12	41	45	11	2.0	3.7	0.2	
C_0040	Betaine aldehyde_+H_2O	249		2.0	0.4	1.2	1.5	0.7	0.8	0.2	0.4	0.5	1.5	1.2	0.6	0.7	0.5	1.7	0.2	
A_0127	cAMP	6076	HMDB0000058	N.D.	N.D.	1.0	N.D.	N.D.	N.D.	N.D.	N.D.	N.D.	N.D.	1.0	N.A.	N.A.	N.A.	1<	N.A.	
C_0158	Carnosine	439224	HMDB0000033	2.9	0.3	28	0.6	0.2	0.5	0.3	0.3	0.05	0.3	6.4	12	0.3	0.2	21.6	0.3	
A_0142	CDP	6132	HMDB0001546	N.D.	N.D.	0.9	N.D.	N.D.	N.D.	N.D.	N.D.	N.D.	N.D.	0.9	N.A.	N.A.	N.A.	1<	N.A.	
A_0129	cGMP	24316	HMDB0001314	N.D.	N.D.	N.D.	N.D.	N.D.	N.D.	N.D.	N.D.	N.D.	N.D.	N.A.	N.A.	N.A.	N.A.	N.A.	N.A.	
C_0024	Choline	305	HMDB0000097	123	27	196	63	55	45	42	30	40	38	93	67	39	5.5	2.4	0.1	
A_0062	*cis* -Aconitic acid	643757	HMDB0000072	2.1	0.8	4.3	1.2	0.7	1.7	1.6	1.7	1.7	1.3	1.8	1.5	1.6	0.2	1.2	0.7	
A_0081	Citric acid	311	HMDB0000094	48	24	113	38	19	34	37	30	35	24	48	38	32	5.2	1.5	0.4	
C_0121	Citrulline	9750	HMDB0000904	8.3	4.8	17	8.7	6.7	9.4	8.7	6.9	6.8	6.7	9.1	4.8	7.7	1.3	1.2	0.6	
A_0124	CMP	6131	HMDB0000095	2.9	2.5	9.8	4.5	3.3	4.6	5.5	3.9	4.5	4.5	4.6	3.0	4.6	0.6	1.0	1.0	
A_0138	CoA_divalent	87642		0.3	1.7	0.3	0.5	0.4	N.D.	N.D.	N.D.	N.D.	N.D.	0.6	0.6	N.A.	N.A.	1<	N.A.	
C_0064	Creatine	586	HMDB0000064	262	62	875	98	71	131	84	60	84	65	273	346	85	28	3.2	0.3	
C_0031	Creatinine	588	HMDB0000562	2.7	0.8	5.1	1.1	1.0	1.9	2.0	2.6	4.1	0.9	2.1	1.8	2.3	1.2	0.9	0.9	
A_0155	CTP	6176	HMDB0000082	N.D.	N.D.	1.6	N.D.	N.D.	N.D.	N.D.	N.D.	N.D.	N.D.	1.6	N.A.	N.A.	N.A.	1<	N.A.	
C_0043	Cys	594	HMDB0000574,HMDB0003417	0.11	N.D.	0.09	N.D.	N.D.	N.D.	0.09	N.D.	0.07	N.D.	0.10	0.02	0.08	0.011	1.2	0.4	
C_0169	Cytidine	6175	HMDB0000089	8.1	1.9	7.1	3.9	2.2	3.0	2.0	1.4	2.0	2.0	4.6	2.9	2.1	0.5	2.2	0.1	
C_0028	Cytosine	597	HMDB0000630	N.D.	N.D.	N.D.	N.D.	N.D.	N.D.	N.D.	N.D.	N.D.	N.D.	N.A.	N.A.	N.A.	N.A.	N.A.	N.A.	
A_0158	dATP	15993	HMDB0001532	N.D.	N.D.	N.D.	N.D.	N.D.	N.D.	N.D.	N.D.	N.D.	N.D.	N.A.	N.A.	N.A.	N.A.	N.A.	N.A.	
A_0153	dCTP	65091	HMDB0000998	N.D.	N.D.	N.D.	N.D.	N.D.	N.D.	N.D.	N.D.	N.D.	N.D.	N.A.	N.A.	N.A.	N.A.	N.A.	N.A.	
A_0056	Dihydroxyacetone phosphate	668	HMDB0001473	13	1.9	25	4.6	2.0	2.4	1.4	1.2	3.6	4.0	9.3	9.9	2.5	1.3	3.7	0.2	
A_0141	dTDP	164628	HMDB0001274	N.D.	N.D.	N.D.	N.D.	N.D.	N.D.	N.D.	N.D.	N.D.	N.D.	N.A.	N.A.	N.A.	N.A.	N.A.	N.A.	
A_0122	dTMP	9700	HMDB0001227	N.D.	N.D.	N.D.	N.D.	N.D.	N.D.	N.D.	N.D.	N.D.	N.D.	N.A.	N.A.	N.A.	N.A.	N.A.	N.A.	
A_0154	dTTP	64968	HMDB0001342	N.D.	N.D.	N.D.	N.D.	N.D.	N.D.	N.D.	N.D.	N.D.	N.D.	N.A.	N.A.	N.A.	N.A.	N.A.	N.A.	
A_0087	Erythrose 4-phosphate	122357	HMDB0001321	N.D.	N.D.	N.D.	N.D.	N.D.	N.D.	N.D.	N.D.	N.D.	N.D.	N.A.	N.A.	N.A.	N.A.	N.A.	N.A.	
A_0128	Fructose 1,6-diphosphate	172313	HMDB0001058	29	0.6	54	6.4	1.1	6.3	1.5	1.4	4.4	4.8	18	23	3.7	2.2	5.0	0.2	
A_0107	Fructose 6-phosphate	603	HMDB0000124	12	0.4	11	2.1	1.4	1.1	N.D.	0.5	1.9	2.4	5.5	5.8	1.5	0.8	3.8	0.2	
A_0012	Fumaric acid	444972	HMDB0000134	13	N.D.	18	9.1	4.0	5.9	8.4	N.D.	6.3	5.8	11	6.0	6.6	1.2	1.7	0.2	
C_0021	GABA	119	HMDB0000112	2.1	1.6	19	2.2	2.2	2.0	2.5	1.0	2.6	1.6	5.3	7.4	1.9	0.7	2.8	0.4	
A_0150	GDP	8977	HMDB0001201	2.1	0.7	6.2	1.8	2.0	1.1	2.1	1.2	1.7	1.2	2.6	2.1	1.5	0.4	1.8	0.3	
C_0089	Gln	738	HMDB0000641,HMDB0003423	240	127	553	180	148	135	155	132	126	144	250	175	139	11	1.8	0.2	
C_0090	Glu	611	HMDB0000148,HMDB0003339	258	116	498	193	139	106	264	112	169	111	241	154	152	67	1.6	0.3	
A_0086	Gluconic acid	10690	HMDB0000625	5.2	3.7	7.6	4.7	2.5	2.6	4.0	3.1	3.9	3.2	4.8	1.9	3.4	0.6	1.4	0.2	
A_0106	Glucose 1-phosphate	65533	HMDB0001586	7.7	0.7	8.6	2.4	1.2	1.8	1.2	1.5	1.4	2.1	4.1	3.7	1.6	0.4	2.6	0.2	
A_0110	Glucose 6-phosphate	5958	HMDB0001401	57	1.8	33	8.8	5.2	0.9	0.9	0.8	6.5	11	21	24	4.1	4.7	5.2	0.2	
C_0205	Glutathione (GSH)	124886	HMDB0000125	16	9.1	25	22	13	0.3	0.2	3.1	1.0	7.1	17	6.4	2.4	2.9	7.1	0.0	**
C_0204	Glutathione (GSSG)_divalent	65359		76	13	167	43	34	32	56	32	40	35	66	61	39	10	1.7	0.4	
C_0006	Gly	750	HMDB0000123	289	65	418	140	94	88	90	79	65	70	201	149	78	11	2.6	0.1	
A_0057	Glyceraldehyde 3-phosphate	729	HMDB0001112	N.D.	N.D.	N.D.	N.D.	N.D.	N.D.	N.D.	N.D.	N.D.	N.D.	N.A.	N.A.	N.A.	N.A.	N.A.	N.A.	
A_0059	Glycerol 3-phosphate	439162	HMDB0000126	210	36	257	114	75	45	70	53	62	61	138	93	58	9.7	2.4	0.1	
A_0002	Glycolic acid	757	HMDB0000115	N.D.	N.D.	N.D.	N.D.	N.D.	N.D.	N.D.	N.D.	N.D.	N.D.	N.A.	N.A.	N.A.	N.A.	N.A.	N.A.	
A_0001	Glyoxylic acid	760	HMDB0000119	N.D.	N.D.	N.D.	N.D.	N.D.	N.D.	N.D.	N.D.	N.D.	N.D.	N.A.	N.A.	N.A.	N.A.	N.A.	N.A.	
A_0135	GMP	6804	HMDB0001397	8.2	7.0	32	13	18	16	27	19	21	18	16	10	20	4.1	0.8	0.4	
A_0161	GTP	6830	HMDB0001273	1.7	N.D.	11	0.7	0.4	N.D.	0.4	N.D.	0.4	N.D.	3.4	5.0	0.4	0.03	9.0	0.3	
C_0093	Guanine	764	HMDB0000132	N.D.	0.02	0.3	0.2	N.D.	0.4	N.D.	N.D.	N.D.	N.D.	0.2	0.15	0.4	N.A.	0.4	N.A.	
C_0195	Guanosine	6802	HMDB0000133	11	1.4	17	5.3	2.7	2.2	1.0	1.2	1.5	1.5	7.5	6.5	1.5	0.5	5.1	0.1	
C_0098	His	773	HMDB0000177	17	6.1	30	11	10	7.9	12	8.7	8.5	9.2	15	9.3	9.2	1.4	1.6	0.2	
C_0039	Homoserine	12647	HMDB0000719	0.3	N.D.	0.5	0.2	N.D.	0.11	N.D.	0.15	0.2	0.15	0.3	0.2	0.14	0.02	2.4	0.2	
C_0057	Hydroxyproline	5810	HMDB0000725	6.8	3.5	16	4.9	3.3	2.4	4.4	3.0	3.3	3.5	6.9	5.3	3.3	0.7	2.1	0.2	
C_0069	Hypoxanthine	790	HMDB0000157	92	30	52	31	24	16	9.3	10	19	23	46	28	16	6.0	2.9	0.1	
C_0061	Ile	791	HMDB0000172	21	7.5	26	13	11	7.7	9.8	7.9	8.8	7.1	16	7.4	8.3	1.1	1.9	0.1	
A_0132	IMP	8582	HMDB0000175	33	8.3	63	19	27	14	28	17	26	44	30	21	26	12	1.2	0.7	
C_0188	Inosine	6021	HMDB0000195	204	56	212	83	50	23	17	19	28	29	121	81	23	5.4	5.2	0.1	
A_0080	Isocitric acid	1198	HMDB0000193	N.D.	N.D.	1.2	N.D.	N.D.	N.D.	1.1	N.D.	1.4	0.9	1.2	N.A.	1.2	0.2	1.1	N.A.	
A_0006	Lactic acid	612	HMDB0000190,HMDB0001311	866	452	2,594	594	396	566	635	484	537	498	981	920	544	60	1.8	0.3	
C_0060	Leu	857	HMDB0000687	39	14	47	24	22	15	20	16	18	16	29	13	17	2.0	1.7	0.1	
C_0083	Lys	866	HMDB0000182,HMDB0003405	40	16	86	26	25	24	33	28	21	28	38	28	27	4.4	1.5	0.4	
A_0027	Malic acid	525	HMDB0000156,HMDB0000744	71	31	74	47	24	36	49	27	47	40	49	23	40	8.9	1.2	0.4	
A_0147	Malonyl CoA_divalent	644066		N.D.	N.D.	N.D.	N.D.	N.D.	N.D.	N.D.	N.D.	N.D.	N.D.	N.A.	N.A.	N.A.	N.A.	N.A.	N.A.	
C_0091	Met	876	HMDB0000696	9.9	4.1	13	6.2	6.6	5.0	6.8	5.7	6.9	6.8	8.1	3.7	6.2	0.9	1.3	0.3	
C_0020	*N,N* -Dimethylglycine	673	HMDB0000092	0.7	0.4	1.0	0.8	0.6	0.7	0.8	0.5	0.5	0.6	0.7	0.2	0.6	0.15	1.1	0.6	
A_0166	NAD^+	5893	HMDB0000902	4.2	0.3	60	1.9	1.0	2.5	2.3	2.2	0.2	0.4	13	26	1.5	1.1	8.8	0.4	
A_0167	$NADP^+$	5886	HMDB0000217	8.6	N.D.	18	2.4	0.6	0.7	1.3	1.0	0.6	0.4	7.5	7.9	0.8	0.3	9.7	0.2	
C_0065	Ornithine	389	HMDB0000214,HMDB0003374	11	5.4	8.3	10	4.8	5.0	7.9	7.1	3.0	6.9	8.0	2.8	6.0	2.0	1.3	0.2	
C_0109	Phe	994	HMDB0000159	21	6.6	23	10	11	7.3	10	8.0	9.4	9.2	14	7.4	8.8	1.1	1.6	0.2	
A_0053	Phosphoenolpyruvic acid	1005	HMDB0000263	9.7	1.6	5.9	6.1	2.6	4.7	5.8	4.1	2.0	2.7	5.2	3.2	3.8	1.6	1.3	0.4	
C_0033	Pro	614	HMDB0000162,HMDB0003411	24	12	38	16	14	17	21	13	17	15	21	11	16	2.9	1.3	0.4	
A_0139	PRPP	7339	HMDB0000280	N.D.	N.D.	0.6	N.D.	N.D.	N.D.	N.D.	N.D.	N.D.	N.D.	0.6	N.A.	N.A.	N.A.	1<	N.A.	
C_0009	Putrescine	1045	HMDB0001414	4.9	0.8	5.7	1.1	0.7	1.1	2.1	1.3	1.4	1.2	2.6	2.4	1.4	0.4	1.9	0.3	
A_0004	Pyruvic acid	1060	HMDB0000243	N.D.	N.D.	N.D.	N.D.	N.D.	N.D.	N.D.	N.D.	N.D.	N.D.	N.A.	N.A.	N.A.	N.A.	N.A.	N.A.	
A_0100	Ribose 5-phosphate	439167	HMDB0001548	14	7.9	4.3	6.7	5.2	3.2	2.9	2.4	2.8	2.8	7.6	3.7	2.8	0.3	2.7	0.0	*
A_0099	Ribulose 5-phosphate	439184	HMDB0000618	91	49	59	46	35	15	17	18	18	18	56	21	17	1.5	3.3	0.0	*
C_0219	*S* -Adenosylmethionine	34755	HMDB0001185	1.3	0.4	4.7	0.9	0.5	0.5	0.9	0.4	0.6	0.4	1.6	1.8	0.5	0.2	2.9	0.3	
C_0012	Sarcosine	1088	HMDB0000271	15	0.8	143	1.3	N.D.	0.3	N.D.	0.4	N.D.	0.5	40	69	0.4	0.11	94.0	0.3	
A_0115	Sedoheptulose 7-phosphate	165007	HMDB0001068	13	2.0	7.8	9.1	7.1	2.6	4.4	3.4	3.7	4.4	7.9	4.1	3.7	0.8	2.1	0.1	
C_0025	Ser	617	HMDB0000187,HMDB0003406	80	32	112	50	40	33	40	37	36	43	63	33	38	3.6	1.7	0.2	
C_0082	Spermidine	1102	HMDB0001257	10	11	14	9.0	2.9	3.7	5.5	5.8	2.0	10	9.4	4.0	5.5	3.1	1.7	0.1	
C_0142	Spermine	1103	HMDB0001256	4.7	8.9	6.3	5.5	2.4	1.3	1.4	3.3	0.9	6.3	5.6	2.4	2.6	2.2	2.1	0.1	
A_0016	Succinic acid	1110	HMDB0000254	25	15	72	29	24	20	37	22	21	14	33	22	23	8.5	1.4	0.4	
C_0038	Thr	6288	HMDB0000167	46	19	91	33	28	23	27	28	26	26	43	28	26	2.1	1.7	0.2	
C_0168	Thymidine	5789	HMDB0000273	0.3	0.3	0.5	0.4	0.3	0.8	0.5	0.3	0.2	0.3	0.4	0.10	0.4	0.2	0.8	0.6	
C_0050	Thymine	1135	HMDB0000262	N.D.	N.D.	N.D.	N.D.	N.D.	N.D.	N.D.	N.D.	N.D.	N.D.	N.A.	N.A.	N.A.	N.A.	N.A.	N.A.	
C_0145	Trp	1148	HMDB0000929	5.8	1.0	9.1	4.7	4.6	4.0	5.5	4.6	4.7	4.5	5.0	2.9	4.7	0.6	1.1	0.8	
C_0126	Tyr	1153	HMDB0000158	17	5.9	22	12	10	8.5	10	9.5	9.5	9.4	13	6.2	9.5	0.7	1.4	0.2	
C_0073	Tyramine	5610	HMDB0000306	0.06	0.06	0.11	0.08	0.08	0.05	0.06	0.03	0.05	0.06	0.08	0.02	0.05	0.012	1.5	0.1	
A_0143	UDP	6031	HMDB0000295	0.4	N.D.	3.8	0.3	0.2	0.2	0.4	N.D.	0.2	0.2	1.1	1.7	0.2	0.07	4.6	0.4	
A_0125	UMP	6030	HMDB0000288	7.8	7.8	31	10	11	16	23	17	17	16	14	9.6	18	2.7	0.8	0.4	
C_0030	Uracil	1174	HMDB0000300	11	5.4	7.8	8.1	8.3	5.2	5.7	4.7	5.9	6.1	8.1	2.0	5.5	0.6	1.5	0.0	*
C_0170	Uridine	6029	HMDB0000296	34	14	29	22	15	11	11	9.3	11	10	23	8.7	10	0.6	2.2	0.0	*
A_0156	UTP	6133	HMDB0000285	0.2	N.D.	4.4	N.D.	N.D.	N.D.	N.D.	N.D.	N.D.	N.D.	2.3	3.0	N.A.	N.A.	1<	N.A.	

C_0035	Val	1182	HMDB0000883	38	18	53	29	26	20	24	20	18	19	33	14	20	2.3	1.6	0.1
C_0010	β-Ala	239	HMDB0000056	8.0	3.9	27	5.1	4.9	2.4	4.6	3.0	3.1	3.5	9.7	9.6	3.3	0.8	3.0	0.2

N.A., Not Available; N.D., Not Detected

¶ The ratio of the detected mean values between the two groups.

|| Welch's t-test (*p<0.05, **p<0.01, ***p<0.001)
